# Computed Tomography as a Characterization Tool for Engineered Scaffolds with Biomedical Applications

**DOI:** 10.3390/ma14226763

**Published:** 2021-11-10

**Authors:** Elena Olăreț, Izabela-Cristina Stancu, Horia Iovu, Andrada Serafim

**Affiliations:** 1 Advanced Polymer Materials Group, University Politehnica of Bucharest, 011061 Bucharest, Romania; olaretelena@gmail.com (E.O.); izabela.stancu@upb.ro (I.-C.S.); horia.iovu@upb.ro (H.I.); 2 Faculty of Medical Engineering, University Politehnica of Bucharest, 011061 Bucharest, Romania; 3 Academy of Romanian Scientists, Splaiul Independentei 54, 050085 Bucharest, Romania

**Keywords:** computed tomography, 3D imaging, quantitative analysis, accurate morphometric characterization

## Abstract

The ever-growing field of materials with applications in the biomedical field holds great promise regarding the design and fabrication of devices with specific characteristics, especially scaffolds with personalized geometry and architecture. The continuous technological development pushes the limits of innovation in obtaining adequate scaffolds and establishing their characteristics and performance. To this end, computed tomography (CT) proved to be a reliable, nondestructive, high-performance machine, enabling visualization and structure analysis at submicronic resolutions. CT allows both qualitative and quantitative data of the 3D model, offering an overall image of its specific architectural features and reliable numerical data for rigorous analyses. The precise engineering of scaffolds consists in the fabrication of objects with well-defined morphometric parameters (e.g., shape, porosity, wall thickness) and in their performance validation through thorough control over their behavior (in situ visualization, degradation, new tissue formation, wear, etc.). This review is focused on the use of CT in biomaterial science with the aim of qualitatively and quantitatively assessing the scaffolds’ features and monitoring their behavior following in vivo or in vitro experiments. Furthermore, the paper presents the benefits and limitations regarding the employment of this technique when engineering materials with applications in the biomedical field.

## 1. Introduction

Basing their work on Röntgen’s X-rays [1] and W.H. Oldendorf’s findings regarding the radiodensity discontinuities of two different materials [2], G.H. Hounsfield and A.M. Cormack developed the first functional medical CT scanner [3]. As a medical device, the use of CT scanners raises great concerns regarding the damage caused by the absorbed X-ray. Therefore, the device is equipped with a low tube voltage (approximately 75 KeV [4]) so that low scanning times and adequate slice thickness (usually between 0.4 and 10 mm [5]) result in images with good resolution and sufficient contrast to permit the visualization of different forms of tissue. CT scanners are used for the purposes of diagnosis and control in oncology [6,7], dentistry [8,9] or orthopedics [10].

Although initially exclusively used in the medical field [11], nowadays, CT scanners are being exploited in a variety of nondestructive measurements. However, given the difference between the areas of application, the characteristics of the equipment are also different. Since no live subjects are imaged, the X-ray tube has a much higher voltage (up to 800 kV [12]), the scanning chamber is smaller, and the resolution and accuracy of the generated images can be easily adjusted by moving the object closer to the source [13]. As a result, the field of application for CT scanners has enlarged, comprising metrology [13,14], quality control [15,16], and even forensic studies [17,18,19,20,21,22] or paleontology [23,24,25,26].

Natural materials have been scanned using CT with the aim of using the obtained information for the fabrication of implantable scaffolds with similar structural features [27,28,29]. CT is often coupled with additive manufacturing techniques in order to obtain scaffolds that would ensure the best possible outcome in terms of architecture, mechanical properties, tissue ingrowth, and so on [29,30].

This paper is dedicated to reviewing the use of laboratory micro- and nano-CT in analyzing implantable scaffolds designed for tissue replacement or repair. The present review addresses the matter of CT use, from the material scientists’ point of view, and will consider both qualitative and quantitative analyses performed exclusively for either the scaffolds’ visualization or the evaluation of their behavior in vivo or in vitro.

## 2. Operating Principle of Laboratory CT Equipment

Even though the working principle of a laboratory CT scanner is basically the same as a medical one, some modifications were required for improved performance. A clinical CT is limited by the radiation dose in respect to patient safety, while in CT scanners used in materials characterization, the radiation dose is not limited, high-power radiation being necessary when thick samples made of high X-ray absorbent materials, such as metals, are investigated. Additionally, a higher spatial resolution and accuracy are necessary for small parts with complex microarchitecture. When using laboratory CT scanners, the sample rotates and moves as close as possible to the source (a smaller sample will allow a positioning closer to the source and enables visualization at higher magnification) [13,31]. An X-ray source (fabricated of tungsten, molybdenum, or copper) produces a high-energy electromagnetic beam, usually characterized by a conical shape, which has the advantage (in detriment of fan beam) of capturing a large part of the sample within a single rotation step. The X-rays are transmitted through the sample, the radiation is attenuated as the material absorbs a part of the electromagnetic ray, and a planar detector collects the attenuated radiations as radiography (or a 2D projection image). Before starting the scanning process, the user must set a series of parameters: scanning voltage and intensity, pixel size, number of frames, rotation angle, and exposure time. After a single projection image is recorded (resulting as a mediation of several frames), the sample is rotated with a preset degree and another radiography is recorded in the new position. These two steps are repeated until a complete rotation is reached. For inhomogeneous samples containing at least two materials with significantly different absorptions (e.g., a polymer–metal composite), a 360° scan is recommended, while for single-phase materials, scanning at 180° is considered to be sufficient. Then, using a reconstruction algorithm, the projection images are computed into cross-sectional images or slices, which are further used for visualization and extraction of morphometric parameters (in either 2D or 3D) or for exporting models for 3D printers [30].

**Figure 1:**
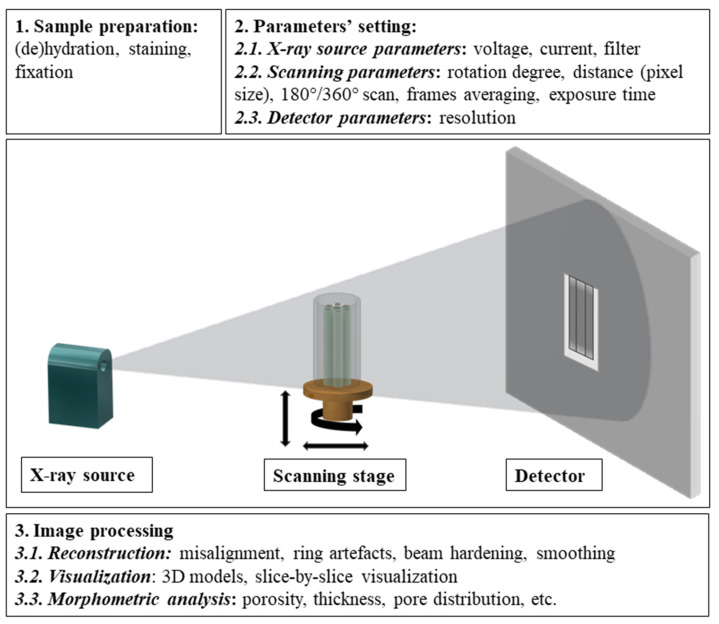
Laboratory CT working principle and main steps for data acquisition, reconstruction, visualization, and analysis.

Some CT scanners provide special material testing stages, which allow the scanning of hydrated samples or the performance of in situ mechanical tests (compression or traction), making it possible to obtain images at different points of deformation [30]. The most popular reconstruction algorithm is filtered back projection (FBP), widely used for fan-beam CT configuration [32]. For cone-beam configuration micro-CTs, the most often used reconstruction algorithm is the Feldkamp-Davis-Kress (FDK) algorithm [33,34], which comprises three main steps: projection space filtering, back projection, and volume space weighting described in [35]. Despite FDK being a nonexact algorithm, it is appreciated for its simplicity, good approximation for small cone angles, and handling data truncated in axial direction [34,36]. FBP or FDK algorithms provide good-quality images in a fast and robust way but require a high amount of X-ray projection data in order to obtain good-quality images and can be affected by image noise [37,38]. Among other types of algorithms, one can find iterative reconstruction algorithms (IR) such as algebraic reconstruction technique (ART) [39] and simultaneous algebraic reconstruction technique (SART) [40,41,42], which allow for obtaining images with comparable quality to FBP, but with lower radiation dose suitable for clinical or in vivo CT [38]. Nevertheless, hybrid algorithms—a combination of FBP and IR—have been developed in order to maximize the benefits of each of them especially for clinical application [37]. The working principle and main steps for acquisition, reconstruction, and analysis are presented in Figure 1.

The quality of the resolution or the acquisition process depends on several factors—X-ray source, detector system, prescanning parameters (flat-field correction, number of frames recorded in each position, degree of rotation, exposure time, X-ray intensity), and pre and postreconstruction processing—but also on several material characteristics (atomic number, thickness). Regardless of the type of analyzed sample, image reconstruction is of paramount importance. Selecting the optimal scanning parameters usually sums up to a trade-off between spatial resolution and noise: a sharp, high-resolution image might also present a lot of noise, and a postscanning smoothing might lead to losing important data. Especially in the case of porous or composite scaffolds, phase segmentation plays an important step and enables, in addition to artifact removal, a clear differentiation of phases (solid particles or voids), which in turn allows a more accurate processing of data and lower errors in subsequent quantitative analyses [43].

Additionally, to obtain optimal results in terms of both 3D reconstructed images and data sets to be used for quantitative measurements, phase contrast performed using conventional CT scanners [44,45,46] or synchrotron radiation sources [47,48,49,50,51] have been largely exploited. While the working principle of CT scanning is based on different X-ray absorbencies within the tested specimen, phase contrast scanning relies on the shift of the X-ray after passing through the scanned object [52]. A thorough study describing the means of obtaining phase contrast using X-rays has been published relatively recently [53]. 

Since CT scanners may be used for the analysis of a multitude of scaffolds—with various architectures or compositions—both pre- and postscanning parameters must be carefully selected, and a customized procedure is necessary for each batch of samples [25].

## 3. CT Imaging of Scaffolds with Biomedical Applications

Most scaffold-based strategies tackled in the regeneration or repair of a damaged organ require a precise control over the microarchitecture of the implanted object. Not only the geometry should perfectly fit the defect, but also the internal structure of the scaffold must be carefully evaluated. The porosity, phase distribution in hybrid or composite materials, and presence of potential defects are of main importance and exhibit a great influence not only on the scaffolds’ mechanical properties and integrity or stability but also on cells’ interactions, nutrients’ diffusion, or the angiogenesis process. 

**Figure 2:**
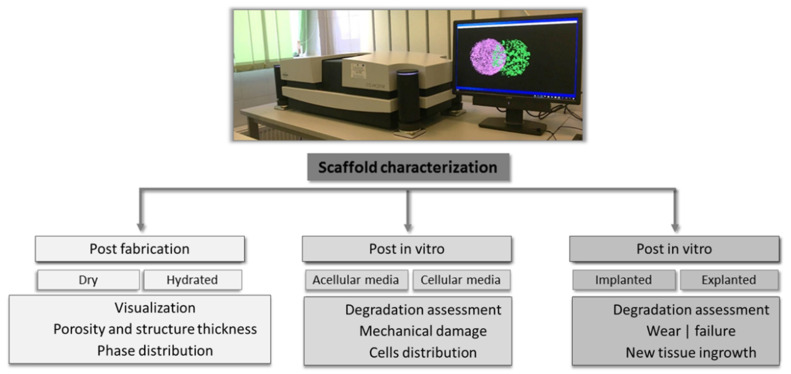
Types of scaffolds’ characterizations and corresponding assessments performed using CT scanners.

A precise imaging of the architectural features is of paramount importance in assessing the behavior of a scaffold in all phases of its evaluation—from fabrication to in vivo testing. Scanning a scaffold to visualize its architectural features is performed in all stages, and the obtained images and data sets may be used in various assessments. As an example, following fabrication, the pores’ distribution or walls’ thickness might be important to assess the homogeneity of a scaffold, while post-in vitro or in vivo testing, the data might offer valuable information regarding the scaffolds’ degradation or integration in the host tissue. Moreover, in vitro tests might be performed in either acellular or cellular conditions, a case in which several parameters might be of interest (e.g., mineralization potential, cells distribution). Regarding the imaging possibilities of visualizing scaffolds without explanting them, to evaluate either their degradation/wear or the tissue ingrowth, in situ imaging is also possible through CT scanning. However, these types of tests are performed using a scanner that resembles the medical CT equipment but is designed to accommodate small animals, such as laboratory rats or rabbits. The X-ray source and detector move around the scanned subject, and the beam’s intensity varies so that it would not hurt the animal, while rendering good-quality images [54]. Considering the significantly different setup of the scanner, these tests are not part of the current review and will not be detailed here. An overview of the types of investigations performed using CT imaging is presented in Figure 2.

Other imaging techniques, such as scanning electron microscopy (SEM) associated with EDAX spectroscopy, atomic force microscopy (AFM), or confocal microscopy (CFM), provide important information regarding surface morphology, topography, and chemical composition. Nonetheless, their use is associated with several drawbacks, such as the need to destroy the sample to obtain suitable geometries that can be further analyzed, and the registered data provide information only with respect to the surface of the sample (SEM, AFM) or thin 3D sections (CFM). For instance, the limitation in terms of imaging depth for SEM is around 200 nm, while CFM, although may penetrate at higher depths (around 100 µm), cannot be applied for opaque scaffolds [55]. CT is used as a complementary technique to obtain both qualitative and quantitative insights regarding the overall internal microarchitecture of the scaffold without causing any alteration to the sample. Its versatility is demonstrated by the possibility to scan all types of materials in hydrated or dried state (i.e., polymers, ceramics, metals, and composites) obtained through various fabrication methods (e.g., membranes, fibers, porous scaffolds, particles). A proper scanning offers not only high-quality images but also a relevant set of data, which will be further used in quantitative determination. Three-dimensional images of samples can reveal information regarding the overall microarchitecture of the scanned object, such as porosity [56,57,58,59], fiber orientation [60], phase distribution [61,62,63,64], the presence and distribution of mineral clusters [65], cell spreading on and within scaffolds [66,67], and effect of degradation evolution on scaffolds’ microstructure [68].

### 3.1. Visualization of Architectural Features through CT Imaging

The visualization of scaffolds’ microarchitecture using CT equipment is an implicit procedure performed after fabrication or following either in vitro or in vivo studies. As opposed to other techniques, CT provides a general overview of either dried or hydrated samples, thus increasing its importance in scaffold characterization for biomedical applications. Despite this significant advantage, only a relatively small number of papers report the evaluation in hydrated state [69,70,71,72] due to the difficulty of obtaining a good X-ray contrast between the hydrated matrix and immersion media.

CT-based evaluation is also widely run for scaffolds fabricated through the 3D printing of a variety of materials, such as natural or synthetic polymers [73,74,75] and polymer-based composites [76,77,78], ceramic powders [79,80,81,82], and glasses [83]. A method article thoroughly describes a micro-CT scanning protocol suitable for Ti6Al4V metal powders with a mean size of 1.4 µm. The article also provides a methodology for image analysis in respect to open and close pore evaluation as well as particle volume, surface area, or sphericity and highlights the importance of standardization analysis for powders used in additive manufacturing (AM) [84]. For scaffolds fabricated through AM, it is of major importance to investigate whether there are any fabrication defects that could lead to mechanical failure [80,85]. One of the most attractive advantages of AM techniques in the field of regenerative medicine is represented by the high reproducibility of the scaffolds’ architectural features and the possibility to progress to the point where customized scaffold designs based on patients’ needs can be fabricated. A computer-assisted design (CAD) software used in AM allows for importing medical CT/NMR files and creating scaffolds suitable as a shape and dimensions for the patient defect [86,87]. Laboratory CT finds its usefulness in determining the fabrication system’s accuracy [88] or quality control for the 3D manufactured scaffold [89,90,91,92] as well as post-in vivo evaluation of the new tissue ingrowth [93,94,95]. 

**Figure 3:**
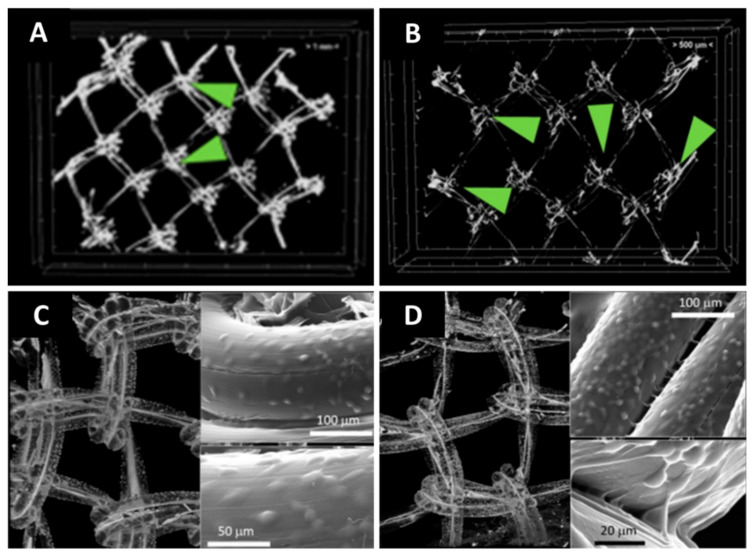
Micro-CT image obtained following coating: (**A**) hydrogel coating on polypropylene mesh following 7 days of testing in acellular conditions, under uniaxial traction (**B**); micro-CT images after cell seeding at 1 (**C**) and 7 (**D**) days, respectively, reproduced after [102] (published under Creative Commons CC BY 4.0 License).

However, the visualization of certain materials is somewhat problematic. Among them, polymers—which are increasingly used in biomedical applications due to their large availability, ease of fabrication, and tunable properties—exhibit low X-ray absorbency. To improve their contrast in CT imaging—in either dry or hydrated state—several staining agents typically used in histology were tested [69,71,96]. Crica et al. used barium chloride, iodine, potassium iodide, and silver nitrate as contrast agents following two staining routes (during and postsynthesis, respectively) for gelatin–poly(vinyl alcohol) scaffolds. Morphology modification and contrast enhancement were observed through SEM and micro-CT, and their results revealed that 1.5 wt.% barium chloride is the ideal amount in order to preserve the initial morphology and improve the image contrast, while iodine staining coupled with hexa(methyl disilazane) fixation proved to be the most advantageous in respect to time efficiency [97]. Collagen-based scaffolds were successfully stained for improved micro-CT imaging in hydrated and dried state using phosphotungstic acid (PTA) [69,71] and Lugol’s solution [98], the results showing minor changes between the two states. Osmium tetroxide-uranyl acetate and uranyl acetate-lead citrate also showed promising results with respect to collagen staining for CT imaging purposes [99], but the time-consuming protocols and the toxicity of the employed reagents must not be disregarded. Protocols for cell labelling were also developed. In this respect, staining agents such as osmium tetroxide [100,101,102] and gold-labeled lectin in tris-buffered saline [103] or ultrasmall superparamagnetic iron oxide nanoparticles [104] were successfully tested. We recently published a comprehensive study regarding the use of natural hydrogels as bioactivators of polypropylene meshes for hernia repair in which micro-CT is used to visualize the uniformity of the hydrogel coating after degradation and cells spreading on the coated meshes [102]. The matter of separating the hydrogel (a natural polymer) from the polypropylene support also presented a certain level of difficulty. Due to the different water affinities of the polymers, we were able to selectively stain the hydrogel through incubation in silver nitrate aqueous solution and thus enhance the contrast between the two materials. Using this protocol, both the coating’s uniformity (Figure 3A) and stability following a week of uniaxial traction performed with continuous PBS perfusion (Figure 3B) were assessed. In addition to optical microscopy and SEM, micro-CT was employed to evaluate the cells’ presence on the coated meshes. The visualization of cells was performed after staining with uranyl acetate, and the registered images offered important information regarding cells spreading on the entire samples (Figure 3C,D). Bosworth et al. used osmium tetroxide to assess the infiltration of mesenchymal stem cells in poly(ε-caprolactone) electrospun yarns [105]. The reported protocol involves the use of glutaraldehyde to fixate the cells, followed by 15 min incubation in 1% aqueous solution of osmium tetroxide in a dark room to increase X-ray contrast of cells on the polymeric scaffold. In another study, chondrocytes seeded on porous gelatin scaffolds were visualized using a protocol involving a combination of silver and gold lysine and a synchrotron radiation based micro-CT equipment [106]. 

Extracellular matrix (ECM) formed following in vitro seeding of mesenchymal stem cells (MSCs) labeled with iron oxide nanoparticles on fibrous polymer-based scaffolds was visualized using synchrotron micro-CT and a semiphase contrast setup [67]. When compared with conventional laboratory CT equipment, this technique presents the advantage of high spatial resolution in the absence of beam hardening effects (due to the use of monochromatic radiation). ECM obtained following in vitro seeding of human-periosteum-derived cells in both static and dynamic conditions on titanium alloy scaffolds was also assessed using Hexabrix and PTA as contrast agents through contrast-enhanced nanofocus CT [107]. Using as comparative techniques Live/Dead viability/cytotoxicity assay and Picrosirius red staining, the study provided not only solid proof of CT reliability in the visualization of ECM in tissue-engineered constructs but also a protocol regarding the processing of the registered images.

**Figure 4:**
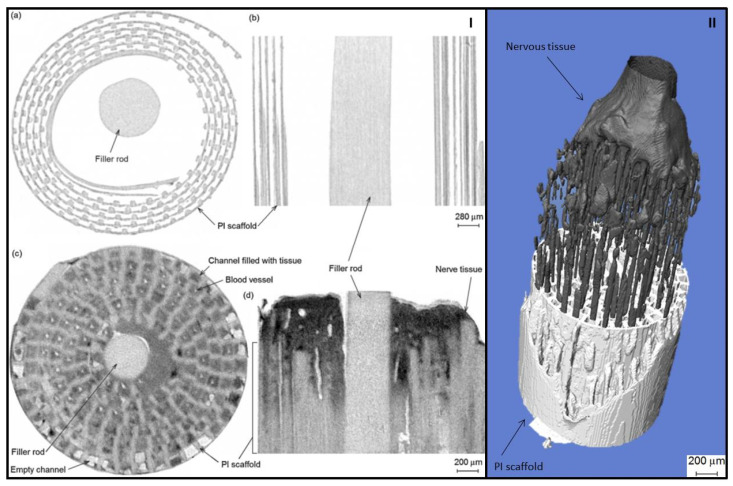
Panel I: µCT (**a**) horizontal cross-section and (**b**) vertical slice of empty nerve scaffold, showing PI scaffold (grey) and empty space (white). (**c**,**d**) show equivalent sections of a nerve scaffold with intergrowing tissue. Osmium stained tissue is dark grey, the PI scaffold is light grey, and empty space (PBS) is white. Tissue has grown down the majority of channels. Blood vessels can be seen as small white circles in the centre of channels (left) or as lines (right); panel II: 3-D model showing a 2 mm length of PI scaffold (white), and the intergrowth of regenerated tissue (black)—the position of the tissue as half of the scaffold is removed. Adapted with permission after [112], copyright Elsevier.

When scanning explanted scaffolds, the drawbacks associated with the use of CT include the difficulty in simultaneously visualizing hard and soft tissues due to bones’ high density [108] and in producing images without beam hardening artifacts when evaluating a metallic scaffold [51,109,110]. Compared with magnetic resonance imaging (MRI), which renders details regarding both mineralized and unmineralized tissues, CT provides high-quality, clear, and reproductive images on the mineral component [111], but a clear delimitation in the mineral phase between a composite scaffold, native bone, and newly formed mineralized tissue is not an easy task due to similar X-ray densities. Visualization of soft tissue is also difficult due to its low density. For an optimal visualization, several compounds based on high atomic number elements and with the ability to bind to soft tissue have been investigated as stains. Watling and collaborators [112] assessed the integration of nervous tissue into a 3D polyimide (PI) scaffold in Lewis rats at 4 weeks postimplantation. The explanted scaffolds were stained with osmium tetroxide (OsO_4_) and subsequently mounted in a pipette tip containing phosphate-buffered saline. The paper demonstrated the successful use of OsO_4_ as an efficient staining agent for micro-CT scanning as it allowed the identification of nervous tissue inside the polymeric scaffold (Figure 4). However, the use of osmium is limited due to its toxicity and strict usage protocol [113].

Another efficient stain for angiogenesis evaluation through CT is Microfil, a radiopaque silicone rubber compound containing lead chromate [114,115]. As opposed to the OsO4 aqueous solution, which stains through diffusion, Microfil is a casting resin that is perfused in the blood vessels and subsequently polymerized. Contrast agents are often futile when investigating the formation of hard tissue upon implantation of a polymeric scaffold due to the high X-ray absorbance of the bone when compared with the organic matrix. As a result, no additional staining must be performed to visualize bone tissue formation [116]. Information regarding the use of contrast agents and staining protocols for improved visualization of solely tissues (in the absence of scaffolds) may be found elsewhere [25,117,118,119].

### 3.2. Determination of 3D Morphometric Parameters

As in nature, when designing scaffolds for regenerative medicine applications, remarkably organized and equilibrated structures are desired to reach optimal functionality. Thus, their microarchitecture exhibits an important impact on mechanical properties, cell attachment, proliferation, and migration, as well as on final tissue ingrowth. Among other characteristics, pore size and total, open/closed porosity, and interconnectivity are pivotal in evaluating the behavior of a material, in either acellular or cellular conditions. It has already been reported that pores with dimensions of 100–300 µm are preferred for cell migration, while for bone ingrowth or capillary formation, larger ones are desired (>300 µm); however, these values vary depending on other factors, such as cell type or tested material [120]. Besides the qualitative evaluation provided by CT, a more comprehensive evaluation is also possible by means of quantitative determination of internal morphometric parameters. These measurements are usually performed with dedicated image processing software. In brief, the cross sections’ reconstruction is followed by several steps: (1) establishing the volume of interest (i.e., the volume of the analyzed sample); (2) binarization: setting a threshold value in respect to which any voxel with a grey value higher than the threshold became white and is considered material, while the rest of the voxels became black and are considered environment/background air; (3) noise filtration, which should be performed before the actual analysis begins [121] (based on a mathematical model, such as median filtration, Gaussian smoothing, and block-matching collaborative filtering [122]).

Representative examples of porous materials analyzed with such protocols are further presented. Porosity determination using micro-CT equipment performed on hydroxyapatite-reinforced polysaccharide scaffolds aimed the assessment of total, closed, and open porosity and pore diameter [123]. Open and total porosities of composite scaffolds based on whey protein and bioactive glass were also evaluated through micro-CT, and the results were compared with mercury intrusion porosimetry. The registered data indicated that there are no significant differences between the two employed methods [124]. In another comparative study, which targeted pore size evaluation, six different methods were used [70]. The pore size was obtained through SEM by averaging the pores’ diameters and micro-CT through 3D morphometric analysis (via sphere-fitting algorithm) and 2D morphometric analysis (via four different algorithms: mean thickness, major diameter, biggest inner circle diameter, and area-equivalent circle diameter). Significant differences were recorded between each method used and indicated that the most effective and advantageous method was 3D morphometric analysis through CT [70]. Liao et al. evaluated the microstructure of a bilayer scaffold observing differences in pore size and an efficient adhesion between the two hydrogels [58]. The internal architecture of the obtained bilayer scaffold was compared with the internal architecture of an articular joint from rabbits, confirming the similarities in terms of microstructure [58]. In alginate-based spongelike structures, micro-CT was employed to evaluate the influence of the composition in the microarchitecture of the synthesized scaffolds in terms of open porosity [125]. Collagen scaffolds’ features in both dry and hydrated states were investigated to understand the fluid flow and hydration mechanisms. An amount of 0.3% PTA in 70% ethanol was used as staining and porosity, and specific surface area and percolation diameter were evaluated to establish the transport pathways through the pore space [69]. Membranes and films were also characterized through CT, revealing characteristic morphostructural features or significant differences in terms of pore size, structure thickness, or specific area between different compositions [126,127,128].

Likewise, materials for bone regeneration have been investigated through CT (e.g., hydroxyapatite scaffolds with different pore architectures [129] or beta-tricalcium phosphate-based materials [130,131]) mainly due to their higher absorption capacity of X-rays, thus obtaining good contrast images. An interesting study presents a comparison of several commercial granules for bone filling defects, with human trabecular bone in terms of porosity, microarchitecture, and molecular composition [132]. Small (250–1000 µm) and large granules (1000–2000 µm) were used to prepare stacks whose microarchitecture was further investigated through micro-CT. It resulted in those small granules generating scaffolds with low interconnected pores (pore diameter around 200 µm), while scaffolds obtained from large granules presented a suitable interconnectivity for osteoconduction (pore diameter above 500 µm) with 3D morphometric parameters similar to human bone [132]. For bone regeneration purposes, CT was performed to evaluate the integration of grafts of natural origin, such as coral grafts [133,134,135] and porcine [136,137,138] or bovine [139,140] bone xerographs. Metal powders were also successfully investigated through micro-CT, despite their higher X-ray absorption capacity [84].

Furthermore, CT characterization renders possible the determination of the degree of anisotropy (DA) and the fractal dimension (FD) of porous structures. Both describe complex structures and are often used in trabecular bone characterization being related to its mechanical strength [141,142,143,144]. A comparison between the applied algorithms (box counting, sausage, and sandbox) to obtain specific parameters (Kolmogorov, Minkowski–Bouligand, and mass–radius FD) of the studied specimens is presented in [145]. While FD describes surface complexity, DA describes the orientation of structures along a certain axis and can be determined, for example, through mean intercept length (MIL) and eigen analysis [146]. These additional parameters (bone volume fraction (BV/TV), trabecular thickness (TbTh), trabecular separation (TbSp), bone surface/bone volume (BS/BV), the connectivity by the Euler number, etc.) provide valuable information regarding bone microstructure [147].

The main advantage of CT in comparison with other techniques is the ability to perform relevant measurements regarding pores’ interconnectivity, because what might appear to be a closed pore in 2D could actually be an open pore in 3D. For a scaffold to be used in tissue regeneration or repair applications, it is imperative to allow cell migration and nutrient diffusion, which are dependent not only on pore size but also on their interconnectivity. While mercury porosimetry and gas pycnometry are not suitable for fragile scaffolds with closed pores [121,148], SEM offers only 2D measurements on small portions of the sample [149]. On the other hand, the advantages of using CT for such measurements has been largely discussed [4,121,148,149], and protocols in this respect have been published, indicating key aspects of data acquisition and analysis. M. Nair et al. recently published a paper that presents a novel analysis method that excludes the effect of scanning artifacts and offers reliable structural parameters [150]. In addition, their work provides ground rules for the selection of the pixel size (prescanning) and volume of interest (in the analysis stage) and offers guidelines on assessing interconnectivity (i.e., percolation diameter and volume interconnectivity) [150].

### 3.3. Phase Distribution

Apart from the visualization of internal morphology and quantitative analysis regarding pores’ size, distribution, and interconnectivity, CT is often employed to evaluate phase distribution in a composite material [64,151]. When such analysis is performed, attention must be paid to the different X-ray adsorption abilities of the two components, which may lead to poor phase contrast. In this respect, synchrotron CTs have proved more efficient than the conventional laboratory CT equipment due to the parallel and monochromatic beam, leading to images free of beam hardening artifacts [152,153]. The superior quality of images obtained through synchrotron CTs is also due to a better signal-to-noise ratio ensured by the high X-ray flux [154,155]. For an optimal visualization of the two phases, two different thresholds must be established so that each phase is identified separately and rendered afterwards together in a color-coded distribution. When analyzing a composite material with two phases that have different X-ray absorption capacities, the segregation of the two phases is relatively simple and is performed by applying two different thresholds in order to identify and quantify each phase separately [62,65]. 

Valuable information regarding the mineral formation within a polymeric matrix may be obtained through CT. In a recent study, the mineralization potential of cuttlebone fish embedded in a pHEMA matrix was assessed following a 2-week incubation in SBF in static vs. dynamic conditions [156]. Although a clear separation between the newly formed mineral and the preloaded phase was not possible in the sample tested in static conditions, the employed micro-CT analysis revealed the formation of new mineral in the cracks provoked by the mechanical stimulation in the continuously compressed sample [156]. The difficulties of separating the new mineral from the preloaded one arise from the similarity between the X-ray absorbencies of the two [157]. Using mineral-coated poly (L-lactic acid) (PLLA) and poly (ε-caprolactone) (PCL), Saito et al. successfully managed to quantify the mineral deposited following implantation [158]. The optimal threshold value for mineral identification was established using the coated polymer samples and subsequently applied to the explanted scaffolds. The registered differences allowed for the calculation of the mineral deposited during the in vivo testing and were in good correlation with the calcium assay performed through the orthocresolphthalein complexone (OCPC) method, thus attesting to the efficiency of this approach in evaluating the mineralization potential of a polymeric scaffold [158].

X-ray adsorption of filaments used in AM for the fabrication of biomedical phantoms can also be assessed through studies regarding the distribution of the radiopaque agent within the polymeric matrix. The distribution of Bi_2_O_3_ in a polylactic acid matrix was investigated, and the additive concentration for radiomimetic CT contrast of the composite filaments was established [159]. 

CT proved its utility in the field of drug delivery as well. Wand et al. used micro-CT to correlate the formulation of the delivery device with its structure and performance. To this end, they investigated the release profile of soluble particulates (gelatin and lactose [160] and gelatin [161], respectively) from a polycaprolactone matrix. The scans, performed prior and after particulates’ dissolution, were used for the quantitative determination of pores’ size and distribution and were further correlated with the release profile of the loaded species [160,161]. In a different study, in addition to Raman and infrared spectroscopy, CT was employed to investigate the internal microstructure of pharmaceutical granules [162]. To this end, PVP-lactose granules were studied, and the CT data (intragranular porosity, excipient and binder volumes) were correlated with the spectrometry results, thus offering a new point of view in the optimization of the granulation process [162]. 

### 3.4. Evaluation of Architectural Features under Mechanical Load

Mechanical properties are part of the main features that dictate the success of a scaffold intended for implantation. In this respect, CT is often used to evaluate when failure during in situ compression or tensile occurs. However, if the equipment is not provided with a mechanical testing stage, samples can be mechanically tested ex situ, and cracks or deformations can be evaluated afterwards. Scaffolds for tissue engineering, metallic [163,164,165], ceramic [81], or polymeric [166,167], mostly designed for bone defects have been mechanically tested and subsequently investigated with the help of CT. 

**Figure 5:**
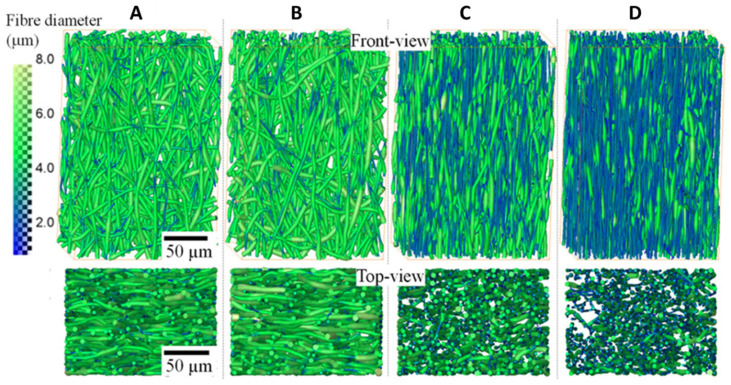
Front and top view 3D rendering of small volumes of the P(3HB-co-4HB) fiber mats and fiber diameter distributions for the unstrained (**A**), 33% (**B**), 193% (**C**), and 352% strained (**D**). The color map on the left represents fiber diameter in micrometers, reproduced after [166] (published under Creative Commons Attribution License).

Ti6Al4V microlattice structures fabricated through laser-based powder bed fusion (LPBF) employing its smallest track width (0.1 mm) were in situ mechanically tested, and their behavior was analyzed using micro-CT. Four loading steps were applied, and images of the tested sample were recorded after each step as well as before testing. It was observed that large deformations and fractures occur at strut joints and that they are dependent on material density. Additionally, surface irregularities appeared due to the small width employed and the limitation of LPBF, but they did not influence the mechanical behavior [168]. The failure mechanism during in situ tensile of electrospun polymeric fibers was also investigated using micro-CT. Starting with random oriented fibers, a first clear observation refers to the reorientation of the fibers along the strain direction, followed by thinning of fiber diameter, localized necking, and failure. Moreover, the fibers’ fusion points remained mostly unstrained until failure, suggesting the strong adhesion between the fibers [166] (Figure 5). An interesting study compares the mechanical behavior of a native glenoid with the mechanical behavior of an implant glenoid mimicking the physiological loading conditions using a loading equipment coupled with micro-CT [169]. However, besides scaffolds used in regenerative medicine, other materials with vast application fields were characterized in this way—rocks [170], cement-based materials [63], composites for wind turbine blades [171], metamaterials [172], and advanced ceramics [173]—even industrial micro-CT was employed for the evaluation of mechanically tested samples [174].

Likewise, another addressed practice that predicts a scaffold’s thermal and mechanical properties as well as deformations under different types of loadings is the use of micro-CT in combination with image-based finite element (FE) simulation [175]. The accuracy of computed FE models in respect to real testing greatly depends on the efficiency in the scaffold’s architecture reproduction. For instance, an ideal 3D model created using CAD technology for 3D printing and the actual 3D-printed scaffold (wherein some additional factors, such as fusion of filaments or porosity within the filament, appear) will exhibit different mechanical behaviors [176]. FE models with high accuracy and complex architecture can be created from micro-CT scans and further imported to dedicated software (e.g., Abaqus) and submerged to FE simulation [177,178,179]. This combination requires more image processing but takes advantage of the high resolution and real microstructure of the scanned scaffold through micro-CT and the possibility to repetitively perform different types of FE simulations using the same 3D model (which is not possible when a real mechanical destructive test is conducted).

### 3.5. Evaluation of Degradation and Tissue Ingrowth

Oftentimes a desired property for scaffolds used in regenerative medicine is biodegradability. Ideally, the healing occurs as the scaffold degrades and is replaced by the newly formed tissue in a synchronized manner [180]. Besides the conventional studies, which usually do not provide information about changes in microstructure, the concurrent degradation–tissue ingrowth process can be assessed using micro-CT imaging [68,181,182].

CT is used in preclinical testing to estimate the stability and integration of a scaffold in the host tissue. Although the technique does not aim to replace other relevant testing methods regarding new tissue ingrowth, such as histology or biochemical characterization, nor other imaging techniques aiming at morphostructural characterization, such as SEM or TEM, CT offers great insight regarding the overall development and organization of new tissue within the context of complex engineered construct implantation. Usually, in addition to CT, other techniques are being employed to accurately evaluate scaffolds’ integration and new bone tissue formation (e.g., SEM or histology) [183]. Histology is one of the most common ones, and although it results in high-quality images at the subcellular level, enabling the visualization of the various types of tissues, this is a time-consuming invasive technique that provides 2D images of a 3D object [51,52]. 

**Figure 6:**
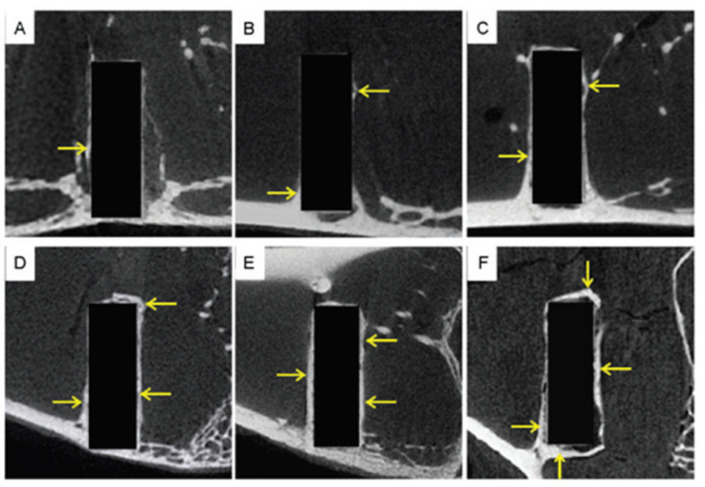
After blanking the previous ROI, a new larger ROI was selected (diameter = 2.5 and length = 6.5 mm) in the same shape and same position to observe new bone formation and assess the stimulatory effects of magnesium alloy on the growth of new bone tissue. New bone formed over time at different rates throughout the experiment. Representative micro-computed tomography images from (**A**) 1, (**B**) 4, (**C**) 12, (**D**) 24, (**E**) 36 and (**F**) 48 weeks are shown. Yellow arrows indicate new bone. Reprinted with permission from [188] (published under Creative Commons Attribution License).

The ability of polymer-based scaffolds to aid the reduction of hard tissue defects was also evaluated using CT. Several research papers report the use of CT as a means to visualize the bone defect before and at various periods of time after the scaffold’s implantation, and not as a characterization technique for the scaffold or the newly formed tissue [183,184]. However, CT was also reported as a tool for the evaluation of the tissue mineral density and was used to obtain images relevant for the assessment of resorbed/deposited bone [185,186]. Aside from polymer-based scaffolds, the integration of metallic implants into bone tissue was also evaluated using fast scans with low resolution realized with a standard laboratory micro-CT [187]. The data sets of the scanned regions were calibrated against the centrally cut histological section of the corresponding sample. Although the registered results proved to be reliable in terms of bone growth assessment within the porous metal implant, in regard to the bone–implant contact area, they did not correlate well with the histomorphometric measurements probably due to the resolution limitation and presence of metal-related artifacts [187]. Concomitant metallic implant degradation and new bone formation were also investigated through CT analysis, and the provided data were used for the in-depth characterization of the bone in terms of bone volume, mineral content, mineral density, and so on [188]. To do so, scans were performed at various pre-established time intervals, and different regions of interest (ROI) were set to assess magnesium pin degradation and bone formation, respectively (Figure 6). As an additional validation technique, implant weight loss was evaluated, but since the results were not consistent—indicating a higher quantity of magnesium corrosion when compared with the volume—the authors considered CT to be “the gold standard” of their study [188]. The influence of the architecture of hydroxyapatite-based 3D-printed scaffolds on their ability to aid bone tissue regeneration was also investigated through micro-CT [189,190,191]. Since hydroxyapatite and bone have similar X-ray absorptions, a proper threshold value that would allow a reliable differentiation between the two had to be established. For this purpose, either a nonimplanted scaffold [189] or Otsu’s method [192] was employed; when vascularization of the newly formed tissue is also being assessed, a staining agent may be used [193]. Evaluations regarding mineralized tissue and bone volume were performed, and the results were corroborated with the information provided by histology [189,190,191,193] or RT-PCR [191,193]. 

### 3.6. Evaluation of Implants’ Wear

**Figure 7:**
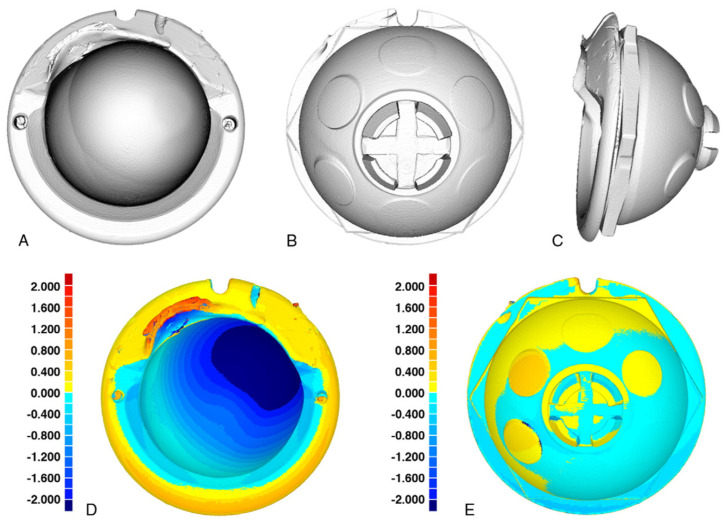
Three-dimensional rendered volume images of the retrieved acetabular liner scanned using micro-CT ((**A**) frontside view, (**B**) backside view, (**C**) isometric view) and 3D surface deviation maps in millimeters ((**D**) frontside view, (**E**) backside view). The liner has been oriented as it was in vivo, with the lip in the superior direction, Reprinted with permission from ref. [200], Elsevier.

The stability of nondegradable implantable prosthetic elements can also be evaluated through CT. Although intensively used, radiography and volumetric and gravimetric measurements employed in such analyses limit the assessment in experimental conditions and fail to deliver reliable data in the case of clinically retrieved prosthetics [194,195] due to the lack of data regarding the geometry or weight before implantation. Furthermore, when these data exist (i.e., when a wear simulator is used), the gravimetric method only offers information on the quantity of lost material, and radiography must be used to approximate the region of the deterioration. CT and coordinate measuring machines (CMMs) use for comparison either idealized unworn geometries of the studied element, obtained through reverse engineering, [194] or matching prosthetics provided by the manufacturer [196]. While CMMs measure the deviation of various points from the surface of the worn sample to provide data [197], CTs rely on high-resolution images obtained following a complete scan of the worn prosthesis [195], offering the possibility to develop surface deviation maps at a resolution considerably higher than the one provided by CMMs [198]. The use of both CT and CMMs has proved to be reliable in offering data regarding global wear volume, local distribution of wear, and creep of retrieval [196,197,199]. Analyzing a retrieved acetabular polyethylene liner, Teeter et al. obtained a 3D map of the surface deviation following nearly 17 years of implantation [200]. Following the scans of both worn and control (an identical unworn element) specimens, the threshold was established, and the reconstructed images were overlapped using a best-fit alignment algorithm. A map of the polyethylene wear was obtained (Figure 7), and an evaluation of the wear penetration rate was computed [200]. In a similar study, the worn tibia inserts were assessed, comparing the data obtained through CT and the gravimetric method for six unworn and six wear-simulated prostheses [198]. The study confirmed the accuracy of the micro-CT data when compared with the gravimetric method and demonstrated the precision of the CT measurements (an error of 0.07% was computed between three scans of the same specimen in different positions) [198]. Engh et al. used micro-CT to map and quantitatively assess the wear of fixed and rotating polyethylene knee platforms using retrieved bearings after 52 months of use [196]. Their study also addressed the matter of the manufacturing tolerances on establishing wear and found that in the short period of use of the analyzed samples, the computed value for the mean wear was less than double the mean difference in tolerance. Furthermore, the study concluded that the wear in the fix-bearing group was double when compared with the rotating-platform group and that less than 70% of the wear accounted for penetration, the rest being due to deformation creep.

Parrilli et al. reported the potential of laboratory micro-CT equipment to be used as a tool for the estimation of wear of ceramic prosthesis elements [201]. The authors established a protocol regarding the scanning, reconstruction, and analysis of a ceramic femoral head. The scanning was performed at two different resolutions using a copper filter and a rotation of 360° of the specimen. In the reconstruction stage, three different beam corrections were applied, resulting in 4 groups of 15 datasets, which were further analyzed. To this end, Otsu’s method was employed to establish a proper threshold, and two levels of despeckle were applied to reduce noise, increasing even further the number of groups to consider to 24, each having 15 datasets. The volume of the sample was computed and compared with the results obtained through the gravimetric method. The large number of gathered data allowed a thorough statistical analysis and the selection of the best protocol for scanning, reconstruction, and assessment of a ceramic prosthesis. More importantly, the paper demonstrated the adequacy of micro-CT in assessing the wear of ceramic elements [201]. 

A summary of the applicability of CT in characterizing scaffold design tissue regeneration and repair is presented in Table 1.

**Table 1:** Summary of CT applicability in characterizing scaffolds with biomedical applications.

Synthetic Scaffolds			
Imaging System	Material| Fabrication Method	Scanning Parameters	Research Objectives and Remarks|References
Micro-CT	PCL (freeze-dried)	8 μm voxel size, 55 kV, 145 mA, 0.3 s integration time	Cell visualization, staining with osmium tetroxide [100]
	PCL (selective laser sintering)	28 μm voxel size, 75 kV, 75 mA	Bone ingrowth evaluation: comparison on compressive modulus obtained through FEA on scanned models and classical compression tests [116]
	PCL (electrospun)	40 kV, power 10 W, 182° scan, 0.61 μm voxel size	Visualization of cell infiltration. Cells were stained in 1% OsO_4_ [105]
	PCL (fused deposition modeling)	Not mentioned	3D visualization before and after in situ tensile tests; morphometric characteristics: porosity, filament thickness, surface area [167]
		16b µm voxel size, 70 kV, 114 µA	Comparison between the 3D CAD model and actual printed scaffold; FE mechanical simulation using micro-CT scans [176]
	Poly (L-lactic acid) and PCL	16 μm pixel size	Scanned before and after implantation; quantification of mineral formation [158]
	Plasma-polymerized allyl amine deposited throughout a porous poly(D,L-lactic acid) scaffold	8 μm voxel size, 55 kV, 145 mA, integration time of 300 ms	Osmium tetroxide-stained cell distribution within the scaffold [101]
	Poly(propylene fumarate)	20.2 µm voxel size, 18 keV	2D, 3D visualization; porosity evaluation through micro-CT and mercury porosimetry [149]
	Poly(propylene fumarate-co-ethylene glycol) (porous scaffold obtained using porogen agents)	10 µm isotropic resolution, 50 Kv, 80 µA, integration time 300 ms	3D visualization and morphometric analysis [181]
	Polyethylene	Resolution 74 µm, 45 kVp, 177 µA	Evaluation of wear of explanted UHMWPE acetabular components; micro-CT proved to be reliable but is limited to a single scan (ex vivo) [195]
		Not mentioned	The wear of an UHMWPE modular acetabular liner was evaluated. A similar unimplanted device was used as reference. The technique to create 3D deviation maps was described [200]
		80 kVp, 450 µA, 92 µm voxel resolution	Evaluation of wear of 24 retrieved tibial polyethylene inserts; imaging of components’ location, penetration, and deformation was possible [196]
		50 µm pixel size, 90 kVp, 40 mA, 0.3° increments with 10 frames averaged per view	Six unworn and six wear-simulated polyethylene tibial inserts were evaluated. No remarkable differences were registered between micro-CT results and gravimetric volume measurements [198]
	Polyimide (PI) (microfabrication)	2.7 µm pixel size, 33 kVp, 197 µA, exposure time 1904 ms, 0.5 mm Al filter	Sciatic nerve visualization within the polymeric scaffold; staining with OsO_4_ [112]
Nano-CT	Poly(ethylene glycol)/poly(ethylene oxide) (electrospinning)	pixel size of 0.5 μm, 30 kV, 450 μA, rotation step of 0.1°, frames averaging 4, exposure time 1300 ms	3D visualization and porosity evaluation [107]
Synchrotron micro-CT	Polyethylene terephthalate multifilament yarns.	0.7 µm pixel width, f photon energy 14.5 keV	Comparison between cell visualization with SR micro-CT and confocal laser scanning microscopy. Samples were stained with gold-labeled lectin [103]
	Poly(3-hydroxybutyrate-co-4-hydroxybutyrate) fiber mats (electrospinning)	0.81 µm pixel size, 180° scan, step size of 0.12°, and exposure time from 0.075 to 0.15 ms	3D visualization and morphometric characterization; in situ tensile tests [166]
**Natural Structures**			
**Imaging System**	**Material|** **Fabrication Method**	**Scanning Parameters**	**Research Objectives and Remarks|References**
Micro-CT	Collagen (freeze-dried)	4 μm pixel size	Scanned in dry and hydrated state (0.3% PTA in 70% ethanol, 48 h) [69]
		2.26 μm pixel size, 47 kV, 142 mA, 1.9 s exposure time, 180° scanning with a rotation step of 0.458	Comparison between different staining agents: osmium tetroxide, uranyl acetate, and lead citrate [99]
		1.5 μm pixel size, 25 kV	Describes the optimized workflow for data acquisition and data analysis [150]
	Alginate modified with N-acetylglucosamine (freeze-dried)	200 μA, 50 kV, 3 frame averaging 0.3°, rotation step, 665 ms exposure time	3D visualization and porosity evaluation [125]
	Chitosan/fish gelatin graphene oxide loaded (freeze-dried)	Pixel size 5 μm, 45 kV, 200 µA, rotation step 0.15°, frame averaging 6	Morphostructural characteristics and 3D visualization [151]
	Coral	Not mentioned	Visualization and morphometric characterization [134]
	Adipose and muscle tissue (bacon strips) and mice hind legs	38–42 µm voxel size, 120 kV, 0.15–3 mm Al filter	Comparison between 28 potential staining agents [117]
	Fish specimens	180° rotation, rotation step: 0.25°. Small samples: 90 keV/8 W; pixel size: <500 nm–5 μm; exposure time: 1 ms Large samples: 50 keV/40 W; pixel size: 15 μm to 75 μm; exposure time: 1.6 ms	Comparison of five staining agents (PTA, IKI, I2E, I2M, and OsO_4_). Scanning parameters were set for each sample [118]
	Chick embryo	20–90 kV, 4–8 W, pixel size 500 μm–5 mm	Comparison of staining agents (gallocyanin-chromalum, I_2_, I_2_E, I_2_M, PTA, OsO_4_) and fixatives [119]
	Bio-Oss; porous HAp	8 µm, 80 kV, 100, exposure time 5 s, Al 0.5 mm filter	3D visualization and quantitative characterization; comparisons between blocks and granules [202]
	Osteopure, undecalcified bovine xenograft, Bio-Oss, CopiOs, TCP Dental, TCP Dental HP, KeraOs, TCH, Ca/P synthetic ceramics	4.95 µm, 80 kV, 100 mA, 0.5 mm Al filter, rotation step 0.25°	Comparison between morphometric characteristics of 9 commercial bone substituents and human bone characteristics [132]
	Bone and coral	20 µm voxel	Comparison between morphological characteristics [133]
	Porcine bone, demineralized porcine bone, and demineralized bone matrix	10 μm voxel	3D visualization and porosity; FEA on micro-CT models [136]
	Porcine bone graft, porcine type I collagen/bone graft composite, and biphasic ceramic material (MBCP™)	Not mentioned	Comparison in terms of osteoconduction capacity; quantitative evaluation of new bone formation [138]
	Bio-Oss Collagen, Orthogen	19 μm^3^ voxel size, 50 kV, 800 μA, frame averaging of 6, angular step of 0.8°, 0.5 mm Al filter	Comparison between morphometric characteristics of both grafts; investigation of their behavior when implanted in a bone defect
	Fragments or granules of deproteinized bovine bone mineral	90 kV, 200 µA, isotropic resolution of 17.2 µm, integration time of 500 ms	Comparison between morphometric characteristics of the two types of grafts; evaluation of new bone formation [140]
	Native glenoid and polyethylene glenoid (implant)	150 kV, 160 µA, Isometric resolution of 35 µm; compression: 0.1 mm/min max. force: 750 N	3D visualization and evaluation of 3D deformation of both samples [169]
Synchrotron micro-CT	Gelatin (freeze-dried)	monochromatic X-ray beam at 10 keV, exposure time 0.7–1.2 s	Chondrocyte distribution after Au/Ag staining [106]
Synchrotron micro-CT	Femoral head	7.50 µm voxel size, 26 keV, integration time 1800–2200 ms,	Evaluation of degree and distribution of bone mineral by micro-CT and synchrotron micro-CT [154]
Micro-CT		8 µm voxel size, 0.5 mm Al filter, 70 kV, 114 µA, 250 ms exposure time	
PET/CT system	Type I collagen	20 μm voxel size	Revealed the interconnected porous architecture [96]
Synchrotron—phase contrast—micro-CT	Murine tibia	Photon energy of 21 keV, 18 ms exposure time, soft tissue 0.325 µm voxel size, mineralized tissue 1.3 µm voxel size	3D visualization of intracortical blood vessels; no staining required [108]
**Hybrid and Composite Scaffolds**			
**Imaging System**	**Material|** **Fabrication Method**	**Scanning Parameters**	**Research Objectives and Remarks|References**
Micro-CT	Collagen–poly(DL-lactide) (freeze-dried)	4.5 μm pixel size	Dry state; pore evaluation in comparison with SEM [70]
	Collagen matrix, poly(DL-lactide) nanofibers, calcium phosphate particles, and sodium hyaluronate (freeze-dried)	4 μm pixel size, 60 kV, 166 μA, 0.25 mm Al filter, frame averaging 2, 180° rotation	Scanned in dried and in hydrated state; staining with Lugol’s solution [98]
	Collagen/graphene oxide (freeze-dried)	58 kV, 385 mA	3D visualization; quantification of apatite formation within the polymeric matrix [65]
	Fish skin gelatin/poly(vinyl alcohol) (freeze-dried)	0.61 μm, 50 kV, 199 µA, 5 average frames, 0.34° rotation step	Comparison between different staining agents/protocols—iodine, barium, silver nitrate, and hexa(methyl disilazane) [97]
	Poly(L-lactide-co-ε-caprolactone) (PLCL) and PLCL/β-tricalcium phosphate	5.0–6.0 µm voxel size, 50 kV, 200 µA	Cells were labeled with 50 nm USPIO nanoparticles. Porosity and cell distribution were investigated [104]
	Chitosan–agarose reinforced with nanohydroxyapatite	12 µm voxel size	3D visualization and porosity evaluation [123]
	Whey protein-bioactive glasses doped with Cu^2+^ and Co^2+^	Not mentioned	3D visualization and porosity evaluation; comparison with results obtained through mercury intrusion porosimetry [124]
	PCL/lactose/gelatin particulates	15 µm voxel resolution, of 173 µA, 30 kV	3D visualization and porosity evaluation. Samples scanned before and after dissolution of particulates—correlation with drug delivery evaluation [160]
	PCL and PCL/tricalcium phosphate (selective laser sintering)	70 μm pixel size	Visualization and porosity evaluation; evaluation of new bone formation [157]
	Calcibon^®^ cement/electrospun PCL fiber composites	2.4 μm pixel size, 70 kV, and 85 μA	3D visualization and morphometric analysis; quantification of material degradation after 4 weeks of implantation and new bone formation [182]
	PP meshes coated with methacryloyl derivatives of gelatin (GelMA) and mucin (MuMA)	Coating: 2.75 µm pixel size, 45 kV, 200 µA, rotation step 0.1°; cells: 1.5 µm pixel size, 70 kV, 130 µA, rotation step 0.3°	Visualization of coatings and cells. Coatings were silver-stained, and cells were stained with 0.5% uranyl acetate [102]
	Cellulose acetate (CA) membranes enriched with CNT and GO	Pixel size 0.5 μm, 50 kV, 200 µA, rotation step of 0.1°, exposure time 1200 ms, frame averaging 3	2D and 3D visualization and pore size distribution [128]
	CaP/silk scaffolds (freeze-drying method)	55 kVp X-ray energy setting	Evaluation of new bone formation after 4 weeks of implantation [184]
	2-hydroxyethyl methacrylate/cuttlefish bone	7 µm pixel size, 70 kV, 175 µA, rotation step 0.4°	Visualization of new mineral formation within the polymeric matrix [156]
	Bioactive glass scaffolds gelatin-coated, cross-linked gelatin-coated, and poly(3-hydroxybutyrate-co-3-hydroxyvalerate)	9 µm pixel size, 50 kVp, 200 µA, integration time of 450 ms	3D morphostructural characterization; bone ingrowth evaluation [186]
	Hydroxyapatite/poly(lactic-co-glycolic acid) (3D-printed scaffolds)	Not specified	Micro-CT was influenced by the scattering effect of the metallic implant. Micro-CT images were not able to distinguish marrow space and soft tissue. SEM and histology assessments were also performed [190]
Nano-CT	Chitosan–gelatin biocomposite films reinforced with graphene oxide	3.5 µm pixel size, 5 kV, 200 μA, exposure time 300 ms, rotation step of 0.1°, frames averaging 5	3D visualization [126]
Micro-CT	Polycaprolactone/hydroxyapatite	Pixel 13.8 μm, rotation step 0.2°, exposure time 4 s, Al 0.25 mm filter	Scaffold visualization [78]
Synchrotron phase-contrast X-ray		White X-ray beam with 19 keV peak energy, pixel size 0.9 μm	Cell visualization [78]
**Ceramics**			
**Imaging System**	**Material|** **Fabrication Method**	**Scanning Parameters**	**Research Objectives and Remarks|References**
Micro-CT	Hydroxyapatite	40 kV, 250 µA	3D visualization and porosity evaluation [129]
	Hydroxyapatite (3D-printed scaffolds)	Resolution approx. 20 µm, 3000 ms shutter speed, 1 × 1 bin size	Evaluation of the influence of scaffold design bone ingrowth through micro-CT [189]
	Alumina (Biolox^®^ forte) femoral head	17.5–35 µm pixel size, 90 kV, 278 μA, Cu filter 0.1 mm, rotation step 0.4^0^, averaging frames 3	Mass and volume loss were evaluated through micro-CT and gravimetrical method [201].
		13.98 μm resolution, 100 kV, 100 μA, A Cu (40 μm) + Al (0.5 mm) filter, 360° scan, rotation step of 0.7°, with an exposure time of 310 ms	Scanned in dry and hydrated state. Bone ingrowth evaluation after 6 months of implantation. Comparison with histological results [187]
**Metal & Alloys**			
**Imaging System**	**Material|** **Fabrication Method**	**Scanning Parameters**	**Research Objectives and Remarks|References**
Micro-CT	Ti6Al4V powder (LPBF)	2 μm voxel size, 100 keV, 100 µA	Describes the workflow powder analysis [84]
	Ti6Al4V microlattice structures (LPBF)	4 µm voxel size, 140 kV, 130 µA, 0.5 mm Cu filter, 500 N loading stage	3D visualization before and after in situ compression tests; porosity evaluation [168]
	Ti6Al4V ELI alloy (electron beam melting)	60 µm voxel size, 220 kV, 120 μA	Comparisons between CAD models and 3D-printed scaffolds; effect of internal porosity on mechanical properties [92]
		13.98 μm resolution, 100 kV, 100 μA, A Cu (40 μm) + Al (0.5 mm) filter, 360° scan, rotation step of 0.7°, with an exposure time of 310 ms	Scanned in dry and hydrated state. Bone ingrowth evaluation after 6 months of implantation. Comparison with histological results [187]
	Ti6Al4V	18.5 µm voxel size, 135 kV, 70 μA	Visualization and porosity evaluation before and after mechanical testing
	Titanium	10 µm pixel size	Porosity evaluation before and after compression tests [163]
	Magnesium alloy (micro-arc-oxidized AZ31) femoral condyle	80 kV, 450 µA, 45 µm pixel size, 400 ms exposure time	Evaluation of the degradation of implants and new bone formation. Gravimetric measurements and histological analyses were also performed
	Zirconia foams	Pixel size of 1.5 μm (for 80 pores/inch) foam and 3 μm (for 45 and 60 pores/inch), 100 kV and 100 μA, 360° scan with a rotation step of 0.1°, 0.11 mm Cu filter	3D visualization and morphometric analysis; compressive mechanical properties determined through FE using micro-CT scans [179]
Nano-CT	Ti6Al4V (selective laser melting)	90 kV, 170 µA, 1 mm Al filter and 1 mm Cu filter, exposure time 500 ms	ECM visualization and quantification; staining agents: Hexabrix 320 and PTA [107]

## 4. Limitations and Perspectives of the CT Technique

Since its release to the public in the 1970s, the CT—both the technique and the equipment—has suffered significant changes. What seemed to be unreachable 50 years ago is now common practice: scanning of a 3D object within a reasonable period of time, obtaining high-resolution images that allow the observation of details at less than 5 µm, and even more, using the registered data sets for quantitative measurements [203]. In addition, the fast progress of additive manufacturing technologies and the current focus on personalized medicine are also factors that put pressure on CT scanner producers to constantly improve their products, making them more accurate and user friendly. The obvious benefits of using CT as an advanced characterization equipment seem to be limited only by the relatively high cost of the apparatus and the need to be operated by trained professionals.

The development of new protocols as solutions for various issues encountered when scanning polymer-based scaffolds, hydrated materials, or soft tissue has also been established. To enable an accurate analysis, staining agents have been proposed [69,71,96,97,98]. Still, a universal protocol is unlikely to emerge since the type of sample and its composition dictates the specimen preparation and the parameters for scanning and reconstruction. Furthermore, the image data processing depends a great deal on the users’ ability to perform a proper separation of the object from the surrounding background (binarization) [204]. As a result, the necessity of using complementary techniques, such as SEM, AFM, or histology, to validate the obtained results has yet to be overcome. Due to the lack of generally accepted protocols and standards, the use of additional procedures is required to undoubtedly confirm the results of a study, leading to increased consumption of time and reagents and the use of complicated equipment and protocols. As far as the authors of this review are aware, using solely CT to discuss, for example, the architectural features or mineral composition of a scaffold has not been reported yet.

With respect to in vivo testing, CT proved to be a dependable technique. Not only it offers information about the degradation or integration of a scaffold into the native tissue, but it also allows scanning of the same sample at different time points, thus decreasing the number of sacrificed animals in preclinical trials. 

The advances in the field of CT corroborated with the limitations of animal models for the validation of implantable scaffolds will soon render this technique indispensable in the field of tissue engineering. Time-lapse scanning can be successfully used to observe the behavior of a sample in various processes, such as degradation or integration [155]. Moreover, the great number of high-quality research studies that prove the adequacy of CT in establishing phase distribution, scaffold degradation, or implant wear performed in either in vitro or in vivo conditions demonstrates the potential of this technique to replace the current gold standard for these analyses. The number of studies in which CT is employed as a characterization method for scaffolds with biomedical applications is increasing yearly. A simple search on sciencedirect.com using as the keywords “CT and tissue engineering” generated over three times more articles in 2021 when compared with 2011. Since research topics no longer subscribe to a single domain and emphasis is put on interdisciplinarity, it would be safe to assume that in the following years, CT will become a popular analysis for the in-depth qualitative and quantitative characterization of scaffolds with biomedical applications. 

## References

[1] Röntgen W.C. (1896). On a New Kind of Rays. Nature.

[2] Oldendorf W.H. (1978). The quest for an image of brain: A brief historical and technical review of brain imaging techniques. Neurology.

[3] Hounsfield G.N. (1973). Computerized transverse axial scanning (tomography): I. Description of system. Br. J. Radiol..

[4] Cengiz I.F., Oliveira J.M., Reis R.L. (2018). Micro-CT–a digital 3D microstructural voyage into scaffolds: A systematic review of the reported methods and results. Biomater. Res..

[5] Liguori C., Frauenfelder G., Massaroni C., Saccomandi P., Giurazza F., Pitocco F., Marano R., Schena E. (2015). Emerging clinical applications of computed tomography. Med. Devices Evid. Res..

[6] Cyran C.C., Paprottka P.M., Eisenblätter M., Clevert D.A., Rist C., Nikolaou K., Lauber K., Wenz F., Hausmann D., Reiser M.F. (2014). Visualization, imaging and new preclinical diagnostics in radiation oncology. Radiat. Oncol..

[7] Stieb S., Kiser K., van Dijk L., Livingstone N.R., Elhalawani H., Elgohari B., McDonald B., Ventura J., Mohamed A.S.R., Fuller C.D. (2020). Imaging for Response Assessment in Radiation Oncology: Current and Emerging Techniques. Hematol. Oncol. Clin. N. Am..

[8] Hol C., Hellén-Halme K., Torgersen G., Nilsson M., Møystad A. (2015). How do dentists use CBCT in dental clinics? A Norwegian nationwide survey. Acta Odontol. Scand..

[9] Vandenberghe B. (2020). The crucial role of imaging in digital dentistry. Dent. Mater..

[10] Anderson P.A., Morgan S.L., Krueger D., Zapalowski C., Tanner B., Jeray K.J., Krohn K.D., Lane J.P., Yeap S.S., Shuhart C.R. (2019). Use of Bone Health Evaluation in Orthopedic Surgery: 2019 ISCD Official Position. J. Clin. Densitom..

[11] Villarraga-Gómez H., Herazo E.L., Smith S.T. (2019). X-ray computed tomography: From medical imaging to dimensional metrology. Precis. Eng..

[12] Ametova E., Probst G., Dewulf W., Carmignato S., Dewulf W., Leach R. (2017). X-ray Computed Tomography Devices and Their Components. Industrial X-ray Computed Tomography.

[13] Kruth J.P., Bartscher M., Carmignato S., Schmitt R., De Chiffre L., Weckenmann A. (2011). Computed tomography for dimensional metrology. CIRP Ann.-Manuf. Technol..

[14] Tan Y., Kiekens K., Welkenhuyzen F., Angel J., De Chiffre L., Kruth J.-P., Dewulf W. (2014). Simulation-aided investigation of beam hardening induced errors in CT dimensional metrology. Meas. Sci. Technol..

[15] Pejryd L., Beno T., Carmignato S. (2014). Computed tomography as a tool for examining surface integrity in drilled holes in CFRP composites. Procedia CIRP.

[16] Kastner J., Plank B., Salaberger D., Sekelja J. Defect and Porosity Determination of Fibre Reinforced Polymers by X-ray Computed Tomography. Proceedings of the 2nd International Symposium on NDT in Aerospace.

[17] Cecchetto G., Amagliani A., Giraudo C., Fais P., Cavarzeran F., Montisci M., Feltrin G., Viel G., Ferrara S.D. (2012). MicroCT detection of gunshot residue in fresh and decomposed firearm wounds. Int. J. Leg. Med..

[18] Cecchetto G., Giraudo C., Amagliani A., Viel G., Fais P., Cavarzeran F., Feltrin G., Davide Ferrara S., Montisci M., Ferrara S.D. (2011). Estimation of the firing distance through micro-CT analysis of gunshot wounds. Int. J. Leg. Med..

[19] Pounder D.J., Sim L.J. (2011). Virtual casting of stab wounds in cartilage using micro-computed tomography. Am. J. Forensic Med. Pathol..

[20] Thali M.J., Taubenreuther U., Karolczak M., Braun M., Brueschweiler W., Kalender W.A., Dirnhofer R. (2003). Forensic microradiology: Micro-computed tomography (Micro-CT) and analysis of patterned injuries inside of bone. J. Forensic Sci..

[21] Sakuma A., Saitoh H., Suzuki Y., Makino Y., Inokuchi G., Hayakawa M., Yajima D., Iwase H. (2013). Age estimation based on pulp cavity to tooth volume ratio using postmortem computed tomography images. J. Forensic Sci..

[22] Azmi N.A., Heo C.C., Shafini N., Mahmud M. (2019). Age estimation of forensically important *blowfly*, *Chrysomya megacephala* (Diptera: *Calliphoridae*) pupae using micro-computed tomography imaging. Trop. Biomed..

[23] Rahman I.A., Adcock K., Garwood R.J. (2012). Virtual Fossils: A New Resource for Science Communication in Paleontology. Evol. Educ. Outreach.

[24] Cunningham J.A., Rahman I.A., Lautenschlager S., Rayfield E.J., Donoghue P.C.J. (2014). A virtual world of paleontology. Trends Ecol. Evol..

[25] Keklikoglou K., Faulwetter S., Chatzinikolaou E., Wils P., Brecko J., Kvaček J., Metscher B., Arvanitidis C. (2019). Micro-computed tomography for natural history specimens: A handbook of best practice protocols. Eur. J. Taxon..

[26] Lewis D. (2019). The fight for control over virtual fossils. Nature.

[27] Drol C.J., Kennedy E.B., Hsiung B.K., Swift N.B., Tan K.T. (2019). Bioinspirational understanding of flexural performance in hedgehog spines. Acta Biomater..

[28] du Plessis A., Broeckhoven C., Yadroitsev I., Yadroitsava I., le Roux S.G. (2018). Analyzing nature’s protective design: The glyptodont body armor. J. Mech. Behav. Biomed. Mater..

[29] Frank M.B., Naleway S.E., Wirth T.S., Jung J.Y., Cheung C.L., Loera F.B., Medina S., Sato K.N., Taylor J.R.A., McKittrick J. (2016). A protocol for bioinspired design: A ground sampler based on sea urchin jaws. J. Vis. Exp..

[30] Helguero C.G., Amaya J.L., Komatsu D.E., Pentyala S., Mustahsan V., Ramirez E.A., Kao I. (2017). Trabecular Scaffolds’ Mechanical Properties of Bone Reconstruction Using Biomimetic Implants. Procedia CIRP.

[31] Rutty G.N., Brough A., Biggs M.J.P., Robinson C., Lawes S.D.A., Hainsworth S.V. (2013). The role of micro-computed tomography in forensic investigations. Forensic Sci. Int..

[32] Özge Onur T. (2021). An application of filtered back projection method for computed tomography images. Int. Rev. Appl. Sci. Eng..

[33] Feldkamp L.A., Davis L.C., Kress J.W. (1984). Practical cone-beam algorithm. J. Opt. Soc. Am. A.

[34] Li H., Zhang H., Tang Z., Hu G. (2008). Micro-computed tomography for small animal imaging: Technological details. Prog. Nat. Sci..

[35] Mueller K., Xu F., Neophytou N., Bouman C.A., Miller E.L., Pollak I. (2007). Why do commodity graphics hardware boards (GPUs) work so well for acceleration of computed tomography. Computational Imaging V.

[36] Brasse D., Humbert B., Mathelin C., Rio M.-C., Guyonnet J.-L. (2005). Towards an inline reconstruction architecture for micro-CT systems. Phys. Med. Biol..

[37] Geyer L.L., Schoepf U.J., Meinel F.G., Nance J.W., Bastarrika G., Leipsic J.A., Paul N.S., Rengo M., Laghi A., De Cecco C.N. (2015). State of the Art: Iterative CT Reconstruction Techniques. Radiology.

[38] Ketola J.H., Karhula S.S., Finnilä M.A.J., Korhonen R.K., Herzog W., Siltanen S., Nieminen M.T., Saarakkala S. (2018). Iterative and discrete reconstruction in the evaluation of the rabbit model of osteoarthritis. Sci. Rep..

[39] Gordon R., Bender R., Herman G.T. (1970). Algebraic Reconstruction Techniques (ART) for three-dimensional electron microscopy and X-ray photography. J. Theor. Biol..

[40] Andersen A. (1984). Simultaneous Algebraic Reconstruction Technique (SART): A superior implementation of the ART algorithm. Ultrason. Imaging.

[41] Ji D., Qu G., Liu B. (2016). Simultaneous algebraic reconstruction technique based on guided image filtering. Opt. Express.

[42] Pang W.-M., Qin J., Lu Y., Xie Y., Chui C.-K., Heng P.-A. (2011). Accelerating simultaneous algebraic reconstruction technique with motion compensation using CUDA-enabled GPU. Int. J. Comput. Assist. Radiol. Surg..

[43] Iassonov P., Gebrenegus T., Tuller M. (2009). Segmentation of X-ray computed tomography images of porous materials: A crucial step for characterization and quantitative analysis of pore structures. Water Resour. Res..

[44] Zhang L., Luo S. (2012). Micro Soft Tissues Visualization Based on X-ray Phase-Contrast Imaging. Open Med. Inform. J..

[45] Yu H., VandeVord P.J., Mao L., Matthew H.W., Wooley P.H., Yang S.Y. (2009). Improved tissue-engineered bone regeneration by endothelial cell mediated vascularization. Biomaterials.

[46] Jones A.C., Arns C.H., Sheppard A.P., Hutmacher D.W., Milthorpe B.K., Knackstedt M.A. (2007). Assessment of bone ingrowth into porous biomaterials using MICRO-CT. Biomaterials.

[47] Neldam C.A., Lauridsen T., Rack A., Lefolii T.T., Jørgensen N.R., Feidenhans’L R., Pinholt E.M. (2015). Application of high resolution synchrotron micro-CT radiation in dental implant osseointegration. J. Cranio-Maxillofac. Surg..

[48] Pratt I.V., Belev G., Zhu N., Chapman L.D., Cooper D.M.L. (2015). In vivo imaging of rat cortical bone porosity by synchrotron phase contrast micro computed tomography. Phys. Med. Biol..

[49] Ma S., Boughton O., Karunaratne A., Jin A., Cobb J., Hansen U., Abel R. (2016). Synchrotron Imaging Assessment of Bone Quality. Clin. Rev. Bone Miner. Metab..

[50] Matsumoto T., Shimizu R., Uesugi K. (2020). In vivo monitoring of bone microstructure by propagation-based phase-contrast computed tomography using monochromatic synchrotron light. Lab. Investig..

[51] Le Cann S., Tudisco E., Turunen M.J., Patera A., Mokso R., Tägil M., Belfrage O., Hall S.A., Isaksson H. (2019). Investigating the mechanical characteristics of bone-metal implant interface using in situ synchrotron tomographic imaging. Front. Bioeng. Biotechnol..

[52] Rawson S.D., Maksimcuka J., Withers P.J., Cartmell S.H. (2020). X-ray computed tomography in life sciences. BMC Biol..

[53] Endrizzi M. (2018). X-ray phase-contrast imaging. Nucl. Instrum. Methods Phys. Res. Sect. A Accel. Spectrometers Detect. Assoc. Equip..

[54] Gabrielson K., Maronpot R., Monette S., Mlynarczyk C., Ramot Y., Nyska A., Sysa-Shah P. (2018). In Vivo Imaging with Confirmation by Histopathology for Increased Rigor and Reproducibility in Translational Research: A Review of Examples, Options, and Resources. ILAR J..

[55] Vielreicher M., Schürmann S., Detsch R., Schmidt M.A., Buttgereit A., Boccaccini A., Friedrich O. (2013). Taking a deep look: Modern microscopy technologies to optimize the design and functionality of biocompatible scaffolds for tissue engineering in regenerative medicine. J. R. Soc. Interface.

[56] Online V.A., Basu B., Swain S.K., Sarkar D. (2013). Cryogenically cured hydroxyapatite–gelatin nanobiocomposite for bovine serum albumin protein adsorption and release. RSC Adv..

[57] Pan T., Song W., Cao X., Wang Y. (2016). 3D Bioplotting of Gelatin/Alginate Scaffolds for Tissue Engineering: Influence of Crosslinking Degree and Pore Architecture on Physicochemical Properties. J. Mater. Sci. Technol..

[58] Liao J., Tian T., Shi S., Xie X., Ma Q., Li G., Lin Y. (2017). The fabrication of biomimetic biphasic CAN-PAC hydrogel with a seamless interfacial layer applied in osteochondral defect repair. Bone Res..

[59] Selaru A., Dragusin D.-M., Olaret E., Serafim A., Steinmüller-Nethl D., Vasile E., Iovu H., Stancu I.-C., Dinescu S., Costache M. (2019). Fabrication and Biocompatibility Evaluation of Nanodiamonds-Gelatin Electrospun Materials Designed for Prospective Tissue Regeneration Applications. Materials.

[60] Shkarina S., Shkarin R., Weinhardt V., Elizaveta M., Kluger P.J., Loza K., Epple M., Ivlev S.I., Baumbach T., Surmeneva M.A. (2018). 3D biodegradable scaffolds of polycaprolactone with silicate-containing hydroxyapatite microparticles for bone tissue engineering: High-resolution tomography and in vitro study. Sci. Rep..

[61] Krause M., Hausherr J.M., Burgeth B., Herrmann C., Krenkel W. (2010). Determination of the fibre orientation in composites using the structure tensor and local X-ray transform. J. Mater. Sci..

[62] Manda M., Oliveira M.B., Mano J.F., Marques A.P., Oliveira J.M., Correlo V.M., Reis R.L. (2012). Gellan Gum-Hydroxyapatite Composite Hydrogels for Bone Tissue Engineering Marianthi. J. Tissue Eng. Regen. Med..

[63] Dumont V.C., Mansur H.S., Mansur A.A.P., Carvalho S.M., Capanema N.S.V., Barrioni B.R. (2016). Glycol chitosan/nanohydroxyapatite biocomposites for potential bone tissue engineering and regenerative medicine. Int. J. Biol. Macromol..

[64] Gupta D., Kocot M., Tryba A.M., Serafim A., Stancu I.C., Jaegermann Z., Pamuła E., Reilly G.C., Douglas T.E.L.L., Maria A. (2020). Novel naturally derived whey protein isolate and aragonite biocomposite hydrogels have potential for bone regeneration. Mater. Des..

[65] Zhou C., Liu S., Li J., Guo K., Yuan Q., Sun S.J. (2018). Collagen Functionalized With Graphene Oxide Enhanced Biomimetic Mineralization and In Situ Bone Defect Repair. ACS Appl. Mater. Interfaces.

[66] Bradley R.S., Robinson I.K., Yusuf M. (2017). 3D X-Ray Nanotomography of Cells Grown on Electrospun Scaffolds. Macromol. Biosci..

[67] Albertini G., Giuliani A., Komlev V., Moroncini F., Pugnaloni A., Pennesi G., Belicchi M., Rubini C., Rustichelli F., Tasso R. (2009). Organization of Extracellular Matrix Fibers Within Polyglycolic Acid–Polylactic Acid Scaffolds Analyzed Using X-Ray Synchrotron-Radiation Phase-Contrast Micro Computed Tomography. Tissue Eng. Part C Methods.

[68] Columbus S., Krishnan L.K., Krishnan V.K. (2014). Relating pore size variation of poly (E-caprolactone) scaffolds to molecular weight of porogen and evaluation of scaffold properties after degradation. J. Biomed. Mater. Res. B Appl. Biomater..

[69] Offeddu G.S., Ashworth J.C., Cameron R.E., Oyen M.L. (2016). Structural determinants of hydration, mechanics and fluid flow in freeze-dried collagen scaffolds. Acta Biomater..

[70] Bartoš M., Suchý T., Foltán R. (2018). Note on the use of different approaches to determine the pore sizes of tissue engineering scaffolds: What do we measure?. Biomed. Eng. Online.

[71] Shepherd J.H., Vriend E.S., Best S.M., Cameron R.E. Analysis of structurally variable lyophilized collagen scaffolds for cell sieving using micro-CT. Proceedings of the Micro-CT User Meeting 2018.

[72] Cecoltan S., Stancu I.-C., Drăguşin D.M., Serafim A., Lungu A., Ţucureanu C., Caraş I., Tofan V.C., Sălăgeanu A., Vasile E. (2017). Nanocomposite particles with improved microstructure for 3D culture systems and bone regeneration. J. Mater. Sci. Mater. Med..

[73] Ji S., Guvendiren M. (2020). 3D Printed Wavy Scaffolds Enhance Mesenchymal Stem Cell Osteogenesis. Micromachines.

[74] Cernencu A.I., Lungu A., Stancu I.C., Serafim A., Heggset E., Syverud K., Iovu H. (2019). Bioinspired 3D printable pectin-nanocellulose ink formulations. Carbohydr. Polym..

[75] Curti F., Drăgușin D.-M., Serafim A., Iovu H., Stancu I.-C. (2021). Development of thick paste-like inks based on superconcentrated gelatin/alginate for 3D printing of scaffolds with shape fidelity and stability. Mater. Sci. Eng. C.

[76] Lai Y., Li Y., Cao H., Long J., Wang X., Li L., Li C., Jia Q., Teng B., Tang T. (2019). Osteogenic magnesium incorporated into PLGA/TCP porous scaffold by 3D printing for repairing challenging bone defect. Biomaterials.

[77] Alexa R.L., Iovu H., Trica B., Zaharia C., Serafim A., Alexandrescu E., Radu I.-C., Vlasceanu G., Preda S., Ninciuleanu C.M. (2021). Assessment of Naturally Sourced Mineral Clays for the 3D Printing of Biopolymer-Based Nanocomposite Inks. Nanomaterials.

[78] Gatto M.L., Furlani M., Giuliani A., Bloise N., Fassina L., Visai L., Mengucci P. (2021). Biomechanical performances of PCL/HA micro- and macro-porous lattice scaffolds fabricated via laser powder bed fusion for bone tissue engineering. Mater. Sci. Eng. C.

[79] Chen G., Sun Y., Lu F., Jiang A., Subedi D., Kong P., Wang X., Yu T., Chi H., Song C. (2019). A three-dimensional (3D) printed biomimetic hierarchical scaffold with a covalent modular release system for osteogenesis. Mater. Sci. Eng. C.

[80] Esposito Corcione C., Gervaso F., Scalera F., Padmanabhan S.K., Madaghiele M., Montagna F., Sannino A., Licciulli A., Maffezzoli A. (2019). Highly loaded hydroxyapatite microsphere/PLA porous scaffolds obtained by fused deposition modelling. Ceram. Int..

[81] Mandal S., Meininger S., Gbureck U., Basu B. (2018). 3D powder printed tetracalcium phosphate scaffold with phytic acid binder: Fabrication, microstructure and in situ X-ray tomography analysis of compressive failure. J. Mater. Sci. Mater. Med..

[82] Farzadi A., Solati-hashjin M., Asadi-eydivand M., Azuan N., Osman A. (2014). Effect of Layer Thickness and Printing Orientation on Mechanical Properties and Dimensional Accuracy of 3D Printed Porous Samples for Bone Tissue Engineering. PLoS ONE.

[83] Zhang J., Chen Y., Xu J., Wang J., Li C., Wang L. (2018). Tissue engineering using 3D printed nano-bioactive glass loaded with NELL1 gene for repairing alveolar bone defects. Regen. Biomater..

[84] Sperling P., Beerlink A., Willie B., Stephan G. (2018). Standard method for microCT-based additive manufacturing quality control 4: Metal powder analysis. MethodsX.

[85] Gong H., Nadimpalli V.K., Rafi K., Starr T., Stucker B. (2019). Micro-CT Evaluation of Defects in Ti-6Al-4V Parts Fabricated by Metal Additive Manufacturing. Technologies.

[86] Landers R., Pfister A., Hübner U., John H., Schmelzeisen R., Mülhaupt R. (2002). Fabrication of soft tissue engineering scaffolds by means of rapid prototyping techniques. J. Mater. Sci..

[87] Grecchi F., Zecca P.A., Macchi A., Mangano A., Riva F., Grecchi E., Mangano C. (2020). Full-digital workflow for fabricating a custom-made direct metal laser sintering (Dmls) mandibular implant: A case report. Int. J. Environ. Res. Public Health.

[88] Gendviliene I., Simoliunas E., Rekstyte S., Malinauskas M., Zaleckas L., Jegelevicius D., Bukelskiene V., Rutkunas V. (2020). Assessment of the morphology and dimensional accuracy of 3D printed PLA and PLA/HAp scaffolds. J. Mech. Behav. Biomed. Mater..

[89] Bartnikowski M., Vaquette C., Ivanovski S. (2020). Workflow for highly porous resorbable custom 3D printed scaffolds using medical grade polymer for large volume alveolar bone regeneration. Clin. Oral Implant. Res..

[90] Wang L., Kang J., Sun C., Li D., Cao Y., Jin Z. (2017). Mapping porous microstructures to yield desired mechanical properties for application in 3D printed bone scaffolds and orthopaedic implants. Mater. Des..

[91] Odeh M., Levin D., Inziello J., Lobo Fenoglietto F., Mathur M., Hermsen J., Stubbs J., Ripley B. (2019). Methods for verification of 3D printed anatomic model accuracy using cardiac models as an example. 3D Print. Med..

[92] Szymczyk P., Hoppe V., Ziółkowski G., Smolnicki M., Madeja M. (2020). The effect of geometry on mechanical properties of Ti6Al4V ELI scaffolds manufactured using additive manufacturing technology. Arch. Civ. Mech. Eng..

[93] Zheng Y., Zhang T., Wei Q., Fan D., Liu X., Li W., Song C., Tian Y., Cai H., Liu Z. (2020). Improved osseointegration with rhBMP-2 intraoperatively loaded in a specifically designed 3D-printed porous Ti6Al4V vertebral implant. Biomater. Sci..

[94] Gao R., Ji W., Xia T., Fan Y., Wei W., Shi L., Liu J., Zhang C., Xue L., Shen J. (2020). Three-dimensional-printed titanium alloy porous scaffold combined with trans-cinnamaldehyde for repairing osteonecrosis of the femoral head in a dog model. Am. J. Transl. Res..

[95] Vidal L., Kampleitner C., Krissian S., Brennan M.Á., Hoffmann O., Raymond Y., Maazouz Y., Ginebra M.-P., Rosset P., Layrolle P. (2020). Regeneration of segmental defects in metatarsus of sheep with vascularized and customized 3D-printed calcium phosphate scaffolds. Sci. Rep..

[96] Zitnay J.L., Reese S.P., Tran G., Farhang N., Bowles R.D., Weiss J.A. (2018). Fabrication of dense anisotropic collagen scaffolds using biaxial compression. Acta Biomater..

[97] Crica L.E., Wengenroth J., Tiainen H., Ionita M., Haugen H.J. (2016). Enhanced X-ray absorption for micro-CT analysis of low density polymers. J. Biomater. Sci. Polym. Ed..

[98] Suchý T., Šupová M., Bartoš M., Sedláček R., Piola M., Soncini M., Fiore G.B., Sauerová P., Kalbáčová M.H., Monika Š. (2018). Dry versus hydrated collagen scaffolds: Are dry states representative of hydrated states?. J. Mater. Sci. Mater. Med..

[99] Faraj K.A., Cuijpers V.M.J.I.J.I., Wismans R.G., Walboomers X.F., Jansen J.A., Van Kuppevelt T.H., Daamen W.F., Walboomers F., Jansen J.A., van Kuppevelt T.H. (2009). Micro-Computed Tomographical Imaging of Soft Biological Materials Using Contrast Techniques. Tissue Eng.-Part C Methods.

[100] Dorsey S.M., Lin-Gibson S., Simon C.G. (2009). X-ray microcomputed tomography for the measurement of cell adhesionand proliferation in polymer scaffolds. Biomaterials.

[101] Barry J.J.A., Howard D., Shakesheff K.M., Howdle S.M., Alexander M.R. (2006). Using a core-sheath distribution of surface chemistry through 3D tissue engineering scaffolds to control cell ingress. Adv. Mater..

[102] Serafim A., Cecoltan S., Olaret E., Dragusin D.-M., Vasile E., Popescu V., Mastalier B.S.M., Iovu H., Stancu I.-C. (2020). Bioinspired Hydrogel Coating Based on Methacryloyl Gelatin Bioactivates Polypropylene Meshes for Abdominal Wall Repair. Polymers.

[103] Thurner P., Müller R., Raeber G., Sennhauser U., Hubbell J.A. (2005). 3D morphology of cell cultures: A quantitative approach using micrometer synchrotron light tomography. Microsc. Res. Tech..

[104] Palmroth A., Pitkänen S., Hannula M., Paakinaho K., Hyttinen J., Miettinen S., Kellomäki M. (2020). Evaluation of scaffold microstructure and comparison of cell seeding methods using micro-computed tomography-based tools. J. R. Soc. Interface.

[105] Bosworth L.A., Rathbone S.R., Bradley R.S., Cartmell S.H. (2014). Dynamic loading of electrospun yarns guides mesenchymal stem cells towards a tendon lineage. J. Mech. Behav. Biomed. Mater..

[106] Zehbe R., Goebbels J., Ibold Y., Gross U., Schubert H. (2010). Three-dimensional visualization of in vitro cultivated chondrocytes inside porous gelatine scaffolds: A tomographic approach. Acta Biomater..

[107] Papantoniou I., Sonnaert M., Geris L., Luyten F.P., Schrooten J., Kerckhofs G. (2014). Three-dimensional characterization of tissue-engineered constructs by contrast-enhanced nanofocus computed tomography. Tissue Eng.-Part C Methods.

[108] Núñez J.A., Goring A., Hesse E., Thurner P.J., Schneider P., Clarkin C.E. (2017). Simultaneous visualisation of calcified bone microstructure and intracortical vasculature using synchrotron X-ray phase contrast-enhanced tomography. Sci. Rep..

[109] Andersson K.M., Nowik P., Persliden J., Thunberg P., Norrman E. (2015). Metal artefact reduction in CT imaging of hip prostheses-an evaluation of commercial techniques provided by four vendors. Br. J. Radiol..

[110] Ejima K., Omasa S., Motoyoshi M., Arai Y., Kai Y., Amemiya T., Yamada H., Honda K., Shimizu N. (2012). Influence of metal artifacts on in vivo micro-CT for orthodontic mini-implants. J. Oral Sci..

[111] Potter K., Sweet D.E., Anderson P., Davis G.R., Isogai N., Asamura S., Kusuhara H., Landis W.J. (2006). Non-destructive studies of tissue-engineered phalanges by magnetic resonance microscopy and X-ray microtomography. Bone.

[112] Watling C.P., Lago N., Benmerah S., FitzGerald J.J., Tarte E., McMahon S., Lacour S.P., Cameron R.E. (2010). Novel use of X-ray micro computed tomography to image rat sciatic nerve and integration into scaffold. J. Neurosci. Methods.

[113] Scheller E.L., Troiano N., Vanhoutan J.N., Bouxsein M.A., Fretz J.A., Xi Y., Nelson T., Katz G., Berry R., Church C.D. (2014). Use of osmium tetroxide staining with microcomputerized tomography to visualize and quantify bone marrow adipose tissue in vivo. Methods Enzymol..

[114] Phelps E.A., Landázuri N., Thulé P.M., Taylor W.R., García A.J. (2010). Bioartificial matrices for therapeutic vascularization. Proc. Natl. Acad. Sci. USA.

[115] Arkudas A., Beier J.P., Pryymachuk G., Hoereth T., Bleiziffer O., Polykandriotis E., Hess A., Gulle H., Horch R.E., Kneser U. (2010). Automatic quantitative micro-computed tomography evaluation of angiogenesis in an axially vascularized tissue-engineered bone construct. Tissue Eng.-Part C Methods.

[116] Williams J.M., Adewunmi A., Schek R.M., Flanagan C.L., Krebsbach P.H., Feinberg S.E., Hollister S.J., Das S. (2005). Bone tissue engineering using polycaprolactone scaffolds fabricated via selective laser sintering. Biomaterials.

[117] Pauwels E., Van Loo D., Cornillie P., Brabant L., Van Hoorebeke L. (2013). An exploratory study of contrast agents for soft tissue visualization by means of high resolution X-ray computed tomography imaging. J. Microsc..

[118] Metscher B.D. (2009). Micro CT for comparative morphology: Simple staining methods allow high-contrast 3D imaging of diverse non-mineralized animal tissues. BMC Physiol..

[119] Metscher B.D. (2009). MicroCT for developmental biology: A versatile tool for high-contrast 3D imaging at histological resolutions. Dev. Dyn..

[120] Bružauskaitė I., Bironaitė D., Bagdonas E., Bernotienė E. (2016). Scaffolds and cells for tissue regeneration: Different scaffold pore sizes—different cell effects. Cytotechnology.

[121] Bertoldi S., Farè S., Tanzi M.C. (2011). Assessment of scaffold porosity: The new route of micro-CT. J. Appl. Biomater. Biomech..

[122] Davidoiu V., Hadjilucas L., Teh I., Smith N.P., Schneider J.E., Lee J. (2016). Evaluation of noise removal algorithms for imaging and reconstruction of vascular networks using micro-CT. Biomed. Phys. Eng. Express.

[123] Kazimierczak P., Palka K., Przekora A. (2019). Development and optimization of the novel fabrication method of highly macroporous chitosan/agarose/nanohydroxyapatite bone scaffold for potential regenerative medicine applications. Biomolecules.

[124] Dziadek M., Douglas T.E.L.L., Dziadek K., Zagrajczuk B., Serafim A., Stancu I.-C.C., Cholewa-Kowalska K. (2020). Novel whey protein isolate-based highly porous scaffolds modified with therapeutic ion-releasing bioactive glasses. Mater. Lett..

[125] Demirbilek M., Türkoğlu Laçin N., Aktürk S. (2017). N-acetylglucoseamine modified alginate sponges as scaffolds for skin tissue engineering. Turk. J. Biol..

[126] Vlasceanu G.M., Crica L.E., Pandele A.M., Ionita M. (2020). Graphene oxide reinforcing genipin crosslinked chitosan-gelatin blend films. Coatings.

[127] Zonderland J., Rezzola S., Gomes D., Espinosa S.C., Lourenço A.H.F., Serafim A., Stancu I.C., Koper D., Liu H., Habibovic P. Full Cell Infiltration and Thick Tissue Formation In Vivo in Tailored Electrospun Scaffolds. https://www.biorxiv.org/content/10.1101/2020.02.19.955948v1.

[128] Ignat S.R., Lazăr A.D., Şelaru A., Samoilă I., Vlăsceanu G.M., Ioniţă M., Radu E., Dinescu S., Costache M. (2019). Versatile biomaterial platform enriched with graphene oxide and carbon nanotubes for multiple tissue engineering applications. Int. J. Mol. Sci..

[129] Tanaka M., Haniu H., Kamanaka T., Takizawa T. (2017). Physico-Chemical, In Vitro, and In Vivo Evaluation of a 3D Unidirectional Porous Hydroxyapatite Scaffold for Bone Regeneration. Materials.

[130] Ndiaye M., Terranova L., Mallet R., Mabilleau G., Chappard D. (2015). Three-dimensional arrangement of b -tricalcium phosphate granules evaluated by microcomputed tomography and fractal analysis. Acta Biomater..

[131] Chappard D., Terranova L., Mallet R., Mercier P. (2015). 3D porous architecture of stacks of β-TcP granules compared with that of trabecular bone: A micro-CT, vector analysis, and compression study. Front. Endocrinol..

[132] Arbez B., Kün-Darbois J.-D., Convert T., Guillaume B., Mercier P., Hubert L., Chappard D. (2019). Biomaterial granules used for filling bone defects constitute 3D scaffolds: Porosity, microarchitecture and molecular composition analyzed by microCT and Raman microspectroscopy. J. Biomed. Mater. Res. B Appl. Biomater..

[133] Puvaneswary S., Balaji Raghavendran H.R., Ibrahim N.S., Murali M.R., Merican A.M., Kamarul T. (2013). A comparative study on morphochemical properties and osteogenic cell differentiation within bone graft and coral graft culture systems. Int. J. Med. Sci..

[134] Cui L., Liu B., Liu G., Zhang W., Cen L., Sun J., Yin S., Liu W., Cao Y. (2007). Repair of cranial bone defects with adipose derived stem cells and coral scaffold in a canine model. Biomaterials.

[135] Matuda Y., Okamura T., Tabata H., Yasui K., Tatsumura M., Kobayashi N., Nishikawa T., Hashimoto Y. (2019). Periodontal regeneration using cultured coral scaffolds in class ii furcation defects in dogs. J. Hard Tissue Biol..

[136] Bracey D.N., Seyler T.M., Jinnah A.H., Lively M.O., Willey J.S., Smith T.L., Van Dyke M.E., Whitlock P.W. (2018). A Decellularized Porcine Xenograft-Derived Bone Scaffold for Clinical Use as a Bone Graft Substitute: A Critical Evaluation of Processing and Structure. J. Funct. Biomater..

[137] Salamanca E., Lee W.-F., Lin C.-Y., Huang H.-M., Lin C.-T., Feng S.-W., Chang W.-J. (2015). A Novel Porcine Graft for Regeneration of Bone Defects. Materials.

[138] Salamanca E., Hsu C.-C., Huang H.-M., Teng N.-C., Lin C.-T., Pan Y.-H., Chang W.-J. (2018). Bone regeneration using a porcine bone substitute collagen composite in vitro and in vivo. Sci. Rep..

[139] Soares M.Q.S., Van Dessel J., Jacobs R., Yaedú R.Y.F., Sant’Ana E., da Silva Corrêa D., Madeira M.F.C., Duarte M.A.H., Rubira-Bullen I.R.F. (2019). Morphometric evaluation of bone regeneration in segmental mandibular bone defects filled with bovine bone xenografts in a split-mouth rabbit model. Int. J. Implant Dent..

[140] Kuchler U., Heimel P., Stähli A., Strauss F.J., Luza B., Gruber R. (2020). Impact of DBBM Fragments on the Porosity of the Calvarial Bone: A Pilot Study on Mice. Materials.

[141] Haire T.J., Hodgskinson R., Ganney P.S., Langton C.M. (1998). A comparison of porosity, fabric and fractal dimension as predictors of the Young’s modulus of equine cancellous bone. Med. Eng. Phys..

[142] Sanchez-Molina D., Velazquez-Ameijide J., Quintana V., Arregui-Dalmases C., Crandall J.R., Subit D., Kerrigan J.R. (2013). Fractal dimension and mechanical properties of human cortical bone. Med. Eng. Phys..

[143] Velázquez-Ameijide J., García-Vilana S., Sánchez-Molina D., Llumà J., Martínez-González E., Rebollo-Soria M.C., Arregui-Dalmases C. (2021). Prediction of mechanical properties of human rib cortical bone using fractal dimension. Comput. Methods Biomech. Biomed. Eng..

[144] Odgaard A. (1997). Three-dimensional methods for quantification of cancellous bone architecture. Bone.

[145] Chappard D., Legrand E., Haettich B., Chalès G., Auvinet B., Eschard J.-P., Hamelin J.-P., Baslé M.-F., Audran M. (2001). Fractal dimension of trabecular bone: Comparison of three histomorphometric computed techniques for measuring the architectural two-dimensional complexity. J. Pathol..

[146] Odgaard A., Kabel J., van Rietbergen B., Dalstra M., Huiskes R. (1997). Fabric and elastic principal directions of cancellous bone are closely related. J. Biomech..

[147] Chappard C., Peyrin F., Bonnassie A., Lemineur G., Brunet-Imbault B., Lespessailles E., Benhamou C.-L. (2006). Subchondral bone micro-architectural alterations in osteoarthritis: A synchrotron micro-computed tomography study. Osteoarthr. Cartil..

[148] Ho S.T., Hutmacher D.W. (2006). A comparison of micro CT with other techniques used in the characterization of scaffolds. Biomaterials.

[149] Moore M.J., Jabbari E., Ritman E.L., Lu L., Currier B.L., Windebank A.J., Yaszemski M.J. (2004). Quantitative analysis of interconnectivity of porous biodegradable scaffolds with micro-computed tomography. J. Biomed. Mater. Res.-Part A.

[150] Nair M., Shepherd J.H., Best S.M., Cameron R.E. (2020). MicroCT analysis of connectivity in porous structures: Optimizing data acquisition and analytical methods in the context of tissue engineering. J. R. Soc. Interface.

[151] Vlasceanu G.M., Selaru A., Dinescu S., Balta C., Herman H., Gharbia S., Hermenean A., Ionita M., Costache M. (2020). Comprehensive Appraisal of Graphene–Oxide Ratio in Porous Biopolymer Hybrids Targeting Bone-Tissue Regeneration. Nanomaterials.

[152] Orhan K., Büyüksungur A. (2020). Fundamentals of Micro-CT Imaging. Micro-Computed Tomography (micro-CT) in Medicine and Engineering.

[153] Wang Y., Garcea S.C., Withers P.J., Beaumont P.W.R., Zweben C.H. (2018). 7.6 Computed Tomography of Composites. Comprehensive Composite Materials II.

[154] Kazakia G.J., Burghardt A.J., Cheung S., Majumdar S. (2008). Assessment of bone tissue mineralization by conventional x-ray microcomputed tomography: Comparison with synchrotron radiation microcomputed tomography and ash measurements. Med. Phys..

[155] Withers P.J., Grimaldi D., Hagen C.K., Maire E., Manley M., Plessis A. (2021). Du X-ray computed tomography. Nat. Rev. Methods Prim..

[156] Dumitrescu G.D., Serafim A., Vasile E., Iovu H., Stancu I.C. (2020). Bioactive biogenous mineral for bone bonding applications. UPB Sci. Bull. Ser. B Chem. Mater. Sci..

[157] Lohfeld S., Cahill S., Barron V., McHugh P., Dürselen L., Kreja L., Bausewein C., Ignatius A. (2012). Fabrication, mechanical and in vivo performance of polycaprolactone/tricalcium phosphate composite scaffolds. Acta Biomater..

[158] Saito E., Suarez-Gonzalez D., Rao R.R., Stegemann J.P., Murphy W.L., Hollister S.J. (2013). Use of Micro-Computed Tomography to Nondestructively Characterize Biomineral Coatings on Solid Freeform Fabricated Poly (L-Lactic Acid) and Poly (ε-caprolactone) scaffolds in vitro and in vivo. Tissue Eng. Part C Methods.

[159] Arnold J., Sarkar K., Smith D. (2020). 3D printed bismuth oxide-polylactic acid composites for radio-mimetic computed tomography spine phantoms. J. Biomed. Mater. Res.-Part B Appl. Biomater..

[160] Wang Y., Wertheim D.F., Jones A.S., Coombes A.G.A. (2010). Micro-CT in drug delivery. Eur. J. Pharm. Biopharm..

[161] Wang Y., Wertheim D.F., Jones A.S., Chang H.-I., Coombes A.G.A. (2010). Micro-CT analysis of matrix-type drug delivery devices and correlation with protein release behaviour. J. Pharm. Sci..

[162] Crean B., Parker A., Roux D.L., Perkins M., Luk S.Y., Banks S.R., Melia C.D., Roberts C.J. (2010). Elucidation of the internal physical and chemical microstructure of pharmaceutical granules using X-ray micro-computed tomography, Raman microscopy and infrared spectroscopy. Eur. J. Pharm. Biopharm..

[163] Arifvianto B., Leeflang M.A., Zhou J. (2017). Diametral compression behavior of biomedical titanium scaffolds with open, interconnected pores prepared with the space holder method. J. Mech. Behav. Biomed. Mater..

[164] Zhang L., Wang S. (2018). Correlation of Materials Property and Performance with Internal Structures Evolvement Revealed by Laboratory X-ray Tomography. Materials.

[165] Khrapov D., Koptyug A., Manabaev K., Léonard F., Mishurova T., Bruno G., Cheneler D., Loza K., Epple M., Surmenev R. (2019). The impact of post manufacturing treatment of functionally graded Ti6Al4V scaffolds on their surface morphology and mechanical strength. J. Mater. Res. Technol..

[166] Maksimcuka J., Obata A., Sampson W.W., Blanc R., Gao C., Withers P.J., Tsigkou O., Kasuga T., Lee P.D., Poologasundarampillai G. (2017). X-ray tomographic imaging of tensile deformation modes of electrospun biodegradable polyester fibers. Front. Mater..

[167] van Kampen K.A., Olaret E., Stancu I.C., Moroni L., Mota C. (2021). Controllable four axis extrusion-based additive manufacturing system for the fabrication of tubular scaffolds with tailorable mechanical properties. Mater. Sci. Eng. C.

[168] Du Plessis A., Kouprianoff D.P., Yadroitsava I., Yadroitsev I. (2018). Mechanical properties and in situ deformation imaging of microlattices manufactured by laser based powder bed fusion. Materials.

[169] Zhou Y., Gong C., Lewis G.S., Armstrong A.D., Du J. (2020). 3D full-field biomechanical testing of a glenoid before and after implant placement. Extrem. Mech. Lett..

[170] Zhou M., Zhang Y., Zhou R., Hao J., Yang J. (2018). Mechanical Property Measurements and Fracture Propagation Analysis of Longmaxi Shale by Micro-CT Uniaxial Compression. Energies.

[171] Wang Y., Mikkelsen L.P., Pyka G., Withers P.J. (2018). Time-lapse helical X-ray computed tomography (CT) study of tensile fatigue damage formation in composites for wind turbine blades. Materials.

[172] Wu W., Qi D., Hu W., Xi L., Sun L., Liao B., Berto F., Qian G., Xiao D. (2020). Synchrotron X-ray micro-computed tomography imaging of 3D re-entrant micro lattice during in situ micro compression experimental process. Mater. Des..

[173] Loa C., Sanob T., Hogan J.D. (2020). Deformation Mechanisms and Evolution of Mechanical Properties in Damaged Advanced Ceramics. J. Eur. Ceram. Soc..

[174] Wang Y., Wang H., Zhou X., Yi X., Xiao Y., Wei X. (2019). In situ X-ray CT investigations of Meso-damage evolution of cemented waste rock-tailings backfill (CWRTB) during triaxial deformation. Minerals.

[175] Kim Y., Yun G.J. (2018). Effects of microstructure morphology on stress in mechanoluminescent particles: Micro CT image-based 3D finite element analyses. Compos. Part A Appl. Sci. Manuf..

[176] Schipani R., Nolan D.R., Lally C., Kelly D.J. (2020). Integrating finite element modelling and 3D printing to engineer biomimetic polymeric scaffolds for tissue engineering. Connect. Tissue Res..

[177] Basri H., Prakoso A.T., Sulong M.A., Md Saad A.P., Ramlee M.H., Agustin Wahjuningrum D., Sipaun S., Öchsner A., Syahrom A. (2020). Mechanical degradation model of porous magnesium scaffolds under dynamic immersion. Proc. Inst. Mech. Eng. Part L J. Mater. Des. Appl..

[178] Biswas P., Guessasma S., Li J. (2020). Numerical prediction of orthotropic elastic properties of 3D-printed materials using micro-CT and representative volume element. Acta Mech..

[179] Askari E., Cengiz I.F., Alves J.L., Henriques B., Flores P., Fredel M.C., Reis R.L., Oliveira J.M., Silva F.S., Mesquita-Guimarães J. (2020). Micro-CT based finite element modelling and experimental characterization of the compressive mechanical properties of 3-D zirconia scaffolds for bone tissue engineering. J. Mech. Behav. Biomed. Mater..

[180] Ding W. (2016). Opportunities and challenges for the biodegradable magnesium alloys as next-generation biomaterials. Regen. Biomater..

[181] Behravesh E., Timmer M.D., Lemoine J.J., Liebschner M.A.K., Mikos A.G. (2002). Evaluation of the in Vitro Degradation of Macroporous Hydrogels Using Gravimetry, Confined Compression Testing, and Microcomputed Tomography. Biomacromolecules.

[182] Yang B., Zuo Y., Zou Q., Li L., Li J., Man Y., Li Y. (2016). Effect of ultrafine poly(ε-caprolactone) fibers on calcium phosphate cement: In vitro degradation and in vivo regeneration. Int. J. Nanomed..

[183] Florczyk S.J., Leung M., Li Z., Huang J.I., Hopper R.A., Zhang M. (2013). Evaluation of three-dimensional porous chitosan-alginate scaffolds in rat calvarial defects for bone regeneration applications. J. Biomed. Mater. Res.-Part A.

[184] Zhang Y., Wu C., Friis T., Xiao Y. (2010). The osteogenic properties of CaP/silk composite scaffolds. Biomaterials.

[185] Weiss P., Obadia L., Magne D., Bourges X., Rau C., Weitkamp T., Khairoun I., Bouler J.M., Chappard D., Gauthier O. (2003). Synchrotron X-ray microtomography (on a micron scale) provides three-dimensional imaging representation of bone ingrowth in calcium phosphate biomaterials. Biomaterials.

[186] Westhauser F., Weis C., Prokscha M., Bittrich L.A., Li W., Xiao K., Kneser U., Kauczor H.U., Schmidmaier G., Boccaccini A.R. (2016). Three-dimensional polymer coated 45S5-type bioactive glass scaffolds seeded with human mesenchymal stem cells show bone formation in vivo. J. Mater. Sci. Mater. Med..

[187] Palmquist A., Shah F.A., Emanuelsson L., Omar O., Suska F. (2017). A technique for evaluating bone ingrowth into 3D printed, porous Ti6Al4V implants accurately using X-ray micro-computed tomography and histomorphometry. Micron.

[188] Xu Y., Meng H., Yin H., Sun Z., Peng J., Xu X., Guo Q., Xu W., Yu X., Yuan Z. (2018). Quantifying the degradation of degradable implants and bone formation in the femoral condyle using micro-CT 3D reconstruction. Exp. Ther. Med..

[189] Simon J.L., Rekow E.D., Thompson V.P., Beam H., Ricci J.L., Parsons J.R., Al S.E.T. (2008). MicroCT analysis of hydroxyapatite bone repair scaffolds created via three-dimensional printing for evaluating the effects of scaffold architecture on bone ingrowth. J. Biomed. Mater. Res.-Part A.

[190] Chang P.C., Luo H.T., Lin Z.J., Tai W.C., Chang C.H., Chang Y.C., Cochran D.L., Chen M.H. (2021). Preclinical evaluation of a 3D-printed hydroxyapatite/poly(lactic-*co*-glycolic acid) scaffold for ridge augmentation. J. Formos. Med. Assoc..

[191] Chang P.-C.C., Luo H.-T.T., Lin Z.-J.J., Tai W.-C.C., Chang C.-H.H., Chang Y.-C.C., Cochran D.L., Chen M.-H.H. (2021). Regeneration of critical-sized mandibular defect using a 3D-printed hydroxyapatite-based scaffold: An exploratory study. J. Periodontol..

[192] Otsu N. (1979). A Threshold Selection Method from Gray-Level Histograms. IEEE Trans. Syst. Man Cybern..

[193] Zhang H., Mao X., Zhao D., Jiang W., Du Z., Li Q., Jiang C., Han D. (2017). Three dimensional printed polylactic acid-hydroxyapatite composite scaffolds for prefabricating vascularized tissue engineered bone: An in vivo bioreactor model. Sci. Rep..

[194] Sagbas B., Numan Durakbasa M. (2012). Measurement of wear in orthopedic prosthesis. Acta Phys. Pol. A.

[195] Bowden A.E., Kurtz S.M., Edidin A.A. (2005). Validation of a micro-CT technique for measuring volumetric wear in retrieved acetabular liners. J. Biomed. Mater. Res.-Part B Appl. Biomater..

[196] Engh C.A., Zimmerman R.L., Hopper R.H., Engh G.A. (2013). Can microcomputed tomography measure retrieved polyethylene wear? Comparing fixed-bearing and rotating-platform knees. Clin. Orthop. Relat. Res..

[197] Uddin M.S., Mak C.Y.E., Callary S.A. (2016). Evaluating hip implant wear measurements by CMM technique. Wear.

[198] Teeter M.G., Naudie D.D.R., McErlain D.D., Brandt J.M., Yuan X., MacDonald S.J., Holdsworth D.W. (2011). In vitro quantification of wear in tibial inserts using microcomputed tomography. Clin. Orthop. Relat. Res..

[199] Yu B., Bradley R.S., Soutis C., Hogg P.J., Withers P.J. (2015). 2D and 3D imaging of fatigue failure mechanisms of 3D woven composites. Compos. Part A Appl. Sci. Manuf..

[200] Teeter M.G., Naudie D.D.R., Charron K.D., Holdsworth D.W. (2010). Three-dimensional surface deviation maps for analysis of retrieved polyethylene acetabular liners using micro-computed tomography. J. Arthroplast..

[201] Parrilli A., Falcioni S., Fini M., Affatato S. (2016). Is micro-computed tomography useful for wear assessment of ceramic femoral heads? A preliminary evaluation of volume measurements. J. Appl. Biomater. Funct. Mater..

[202] Turco G., Porrelli D., Marsich E., Vecchies F., Lombardi T., Stacchi C., Di Lenarda R. (2018). Three-Dimensional Bone Substitutes for Oral and Maxillofacial Surgery: Biological and Structural Characterization. J. Funct. Biomater..

[203] Lin Q., Andrew M., Thompson W., Blunt M.J., Bijeljic B. (2018). Optimization of image quality and acquisition time for lab-based X-ray microtomography using an iterative reconstruction algorithm. Adv. Water Resour..

[204] Bartos M., Suchý T., Tonar Z., Foltán R., Kalbacova M.H. (2018). Micro-CT in tissue engineering scaffolds for bone regeneration: Principles and application. Ceram.-Silik..

